# Glucagon-like peptide-1 receptor agonists for weight management in mental illness: a network meta-analysis

**DOI:** 10.1038/s41398-026-04253-6

**Published:** 2026-07-08

**Authors:** Taro Kishi, Toshikazu Ikuta, Kenji Sakuma, Masakazu Hatano, Takeshi Takayanagi, Atsushi Suzuki, Nakao Iwata

**Affiliations:** 1 Department of Psychiatry, Fujita Health University School of Medicine, Toyoake, Aichi 470-1192 Japan; 2 Department of Communication Sciences and Disorders, School of Applied Sciences, University of Mississippi, University, Oxford, MS 38677 USA; 3 Department of Pharmacotherapeutics and Informatics, Fujita Health University School of Medicine, Toyoake, Aichi 470-1192 Japan; 4 Department of Endocrinology, Diabetes and Metabolism, Fujita Health University, School of Medicine, Toyoake, Aichi 470-1192 Japan

**Keywords:** Schizophrenia, Bipolar disorder

## Abstract

**Background/Objectives:**

This network meta-analysis involved nine randomized controlled trials, comprising 595 participants (average age = 37.8 years, 55.3% female, 83.9% schizophrenia spectrum disorders), evaluating the comparative risk–benefit profiles of glucagon-like peptide-1 receptor agonists in individuals with obesity comorbid with mental illness.

**Subjects/Methods:**

Outcomes included body weight (primary); body mass index (BMI); waist circumference; blood-based measures, including fasting plasma glucose, hemoglobin A1c (HbA1c), total cholesterol, low-density lipoprotein cholesterol, high-density lipoprotein cholesterol, and triglycerides; systolic and diastolic blood pressure; death; all-cause discontinuation; adverse event-related discontinuation; serious adverse events (SAEs), injection site-related adverse events, headache, dizziness, nausea, vomiting, constipation, and diarrhea; psychiatric hospitalization; and changes in overall schizophrenia symptoms.

**Results:**

Treatment arms comprised subcutaneous exenatide (S-EXE)(twice-daily [BID]/once-weekly [QW]), subcutaneous liraglutide (S-LIR)(once-daily [QD]), and subcutaneous semaglutide (S-SEM)(QW), alongside a control group (placebo, k = 8; non-placebo, k = 1). S-LIR(QD) and S-SEM(QW) were significantly associated with reduced body weight compared with control, with standardized mean differences (95% confidence intervals) of −0.945 ( − 1.784 to −0.106) and −2.101 ( − 2.978 to −1.224), respectively. S-LIR(QD) was associated with waist circumference and HbA1c level reductions, lower SAE incidences, and higher nausea, vomiting, and constipation incidences compared with the control. S-SEM(QW) was associated with reduced BMI, waist circumference, fasting plasma glucose, and HbA1c levels, and higher nausea, vomiting, and constipation compared with the control. Each drug did not differ from control regarding other outcomes.

**Conclusions:**

S-SEM(QW) may be the preferred treatment option, given its largest estimated effects versus control on body weight; however, its comparative ranking is uncertain due to sparse networks, substantial heterogeneity, and predominant indirect evidence.

## Introduction

Obesity in individuals with mental illness is a major clinical concern, as its prevalence is substantially higher than in the general population [1, 2]. It contributes to breathlessness through cardiopulmonary impairment; knee and hip pain associated with joint stiffness and range of motion reduction; a constellation of metabolic abnormalities, including dysregulated lipid and glucose metabolism; and dysfunction across multiple organ systems, including the renal, upper airway, nervous, urinary, and reproductive systems [3]. Consequently, it increases the risk of developing diseases, including cardiovascular disease, certain cancers, and type 2 diabetes [3]. In individuals with mental illness, obesity is driven by multiple factors, including the effects of pharmacotherapies (e.g., antipsychotics), reduced daily physical activity, and diminished motivation to maintain physical health associated with psychiatric symptoms [2, 4, 5]. These differences indicate distinct pathophysiology and clinical aspects of obesity between general adults and individuals with mental illness. Recent guidelines for general adults with obesity or overweight recommend adding pharmacological agents—including glucagon-like peptide-1 receptor agonists (GLP-1 RAs)—to lifestyle interventions, rather than continuing lifestyle interventions alone, for those who have had an inadequate response to lifestyle interventions [6, 7]. Meanwhile, recent guidelines for individuals with schizophrenia and comorbid obesity recommend not only lifestyle interventions but also switching to an antipsychotic medication with more favorable metabolic profiles (e.g., aripiprazole), or using adjunctive treatments, including metformin or GLP-1 RAs [1]. Notably, both sets of guidelines recommend the use of GLP-1 RAs. Other adjunctive pharmacological approaches to mitigate antipsychotic related metabolic abnormalities have been evaluated, including melatonin or ramelteon, with double-blind, randomized, placebo-controlled trials (DBRPCTs) indicating modest improvements in metabolic syndrome components [8].

Recently, two meta-analyses of GLP-1 RAs for individuals with obesity comorbid with mental illness have been published [9, 10]. However, these meta-analyses did not include semaglutide—the newest GLP-1 RA and a recommended first-line treatment for individuals with obesity [6, 7, 11–14]. A network meta-analysis of general adults with obesity revealed that semaglutide was associated with decreased weight, normoglycemia restoration, type 2 diabetes remission, reduced heart failure-related hospitalization, and decreased major adverse cardiovascular events and pain in knee osteoarthritis [15]. In 2025, three DBRPCTs of once-weekly subcutaneous semaglutide [S-SEM (QW)] for individuals with obesity comorbid with mental illness were reported [16–18]. Further, previous meta-analyses did not comprehensively assess acceptability, tolerability, and safety outcomes of GLP-1 RAs [9, 10]. Therefore, we conducted an updated network meta-analysis of GLP-1 RAs for individuals with obesity comorbid with mental illness. This network meta-analysis encompassed 24 outcomes associated with efficacy, acceptability, tolerability, and safety (e.g., physical events, including nausea; and mental events, including psychiatric hospitalization and change in psychopathology), thereby providing a broader scope than previous meta-analyses.

## Materials and methods

This study followed the Preferred Reporting Items for Systematic Reviews and Meta-Analyses guidelines [19] (Table S1) and the Cochrane Handbook for Systematic Reviews of Interventions [20] and was registered on the Open Science Framework (https://osf.io/e89uf/overview). At least two authors validated the literature search, data transfer accuracy, and calculations.

### Search strategy and inclusion criteria

The “PICOS” strategy was employed for systematic literature review as follows. “Participants” were adults with obesity comorbid with mental illness; “intervention” consisted of GLP-1 RA treatments at dosages approved for clinical use in the United States [21], Europe [22], or Japan [23] (detailed information regarding the interventions is provided in the “Meta-analysis methods” section); “control” was a placebo, treatment as usual (i.e., no placebo), or other active treatments; “outcomes” were efficacy, acceptability, tolerability, and safety, as detailed in the following section; and “studies” were randomized, controlled trials. Eligible studies comprised articles focusing on adults with any mental illness and comorbid obesity, whereas those involving pediatric or adolescent participants were excluded.

PubMed, Cochrane Library, and Embase databases were searched for studies published until April 28, 2026, without language restrictions. The search terms for PubMed and the Cochrane Library included (“exenatide” OR “liraglutide” OR “lixisenatide” OR “dulaglutide” OR “semaglutide” OR “tirzepatide” OR “albiglutide” OR “orforglipron”) (“schizophrenia” OR “bipolar” OR “depressi*”) AND (“random*”). The search terms for Embase included (“exenatide”/exp OR “exenatide” OR “liraglutide”/exp OR “liraglutide” OR “lixisenatide”/exp OR “lixisenatide” OR “dulaglutide”/exp OR “dulaglutide” OR “semaglutide”/exp OR “semaglutide” OR “tirzepatide”/exp OR “tirzepatide” OR “albiglutide”/exp OR “albiglutide” OR “orforglipron”/exp OR “orforglipron”) AND (“randomized controlled trial”/exp OR “randomized controlled trial”) AND (“schizophrenia”/exp OR “schizophrenia” OR “bipolar disorder”/exp OR “bipolar disorder” OR “depression”/exp OR “depression”). Further, reference lists of the selected research and review articles were manually searched for additional relevant published and unpublished studies, including conference abstracts. Moreover, clinical trial registries (ClinicalTrials.gov [24], EU Clinical Trials Register [25], and the World Health Organization–International Clinical Trials Registry Platform [26]) were searched to ensure the trials were comprehensive and to minimize the effect of publication bias. Any discrepancies in the selected articles were resolved through author consensus. Multiple papers or academic conference abstracts that were reported despite the same research were screened by confirming the clinical trial registration number and/or reference to past review articles [9, 10].

### Outcome measures, data synthesis, and data extraction

Outcomes were body weight (primary); body mass index (BMI); waist circumference; blood-based measures, including fasting plasma glucose, hemoglobin A1c (HbA1c), total cholesterol, low-density lipoprotein cholesterol, high-density lipoprotein cholesterol, and triglycerides; systolic and diastolic blood pressure; death; all-cause discontinuation (treatment acceptability); adverse event-related discontinuation (treatment tolerability); incidences of serious adverse events (SAEs), injection site-related adverse events, headache, dizziness, and gastrointestinal symptoms (e.g., nausea, vomiting, constipation, diarrhea); incidence of psychiatric hospitalization during the trial; and mean change from baseline to endpoint in overall schizophrenia symptom scores as measured with standardized rating scales [27–29]. Table S2 shows details of the data synthesis for the last two outcomes.

To minimize attrition bias, we exclusively used results from intention-to-treat (ITT) or modified ITT populations, rather than relying on completer-only data. This approach ensures that the results better represent the outcomes of the broader randomized population by including participants who discontinued the trial prematurely [20]. Our systematic review employing the Cochrane risk of bias tool for randomized trials version 2 (https://www.riskofbias.info/) assessed the overall risk of bias for each trial.

### Meta-analysis methods

A frequentist network meta-analysis with a random-effects model was conducted [30, 31]. Standardized mean differences (SMDs) were calculated for continuous variables. To improve clinical interpretability, we provided back-transformed mean differences (MDs) alongside the SMDs. According to the methodology recommended in the Cochrane Handbook [20], we calculated the pooled standard deviation (SD) of the placebo groups—excluding non-placebo data—from the studies that used standard units. The SMDs derived from our network meta-analysis were then multiplied by this SD to calculate the MDs. Odd ratios (ORs) were calculated for dichotomous variables. Furthermore, risk differences (RDs) and numbers needed to treat to harm (NNTHs) were calculated only when network meta-analysis revealed significant between-group differences based on ORs. All effect sizes were estimated with their corresponding 95% confidence intervals (CIs). Heterogeneity at both the network and local levels was assessed based on *τ*^*2*^ or *I*^*2*^ statistics. Initially, we planned to assess inconsistency employing the design-by-treatment interaction model and the Separating Indirect from Direct Evidence test (node-splitting model) [32, 33]. The former was implemented; however, the latter was not feasible because of the absence of closed loops and direct head-to-head comparisons between active drugs, which precluded a statistical evaluation of local inconsistency. Surface under the curve cumulative ranking (SUCRA) probabilities were applied to rank treatments for each outcome. To validate the analysis, the sufficiency of the distribution differences was identified by comparing the distribution of possible effect modifiers across included treatments in the network meta-analysis using the Kruskal–Wallis test (continuous variables) and the Pearson chi-square test or Fisher’s exact test (categorical variables) and by evaluating their actual effect on the treatment effect through network meta-regression analyses [34, 35]. In addition to the primary outcome, all-cause discontinuation was included in the network meta-regression and sensitivity analyses. This outcome serves as a proxy for overall treatment acceptability, as it captures the balance between efficacy and safety. Potential confounding factors were the proportion of females, mean age, body weight at baseline, and baseline glycemic status (studies involving patients without prediabetes or dysglycemia vs. those with these conditions). Additional study-level variables were study duration, total number of participants, funding source (industry sponsored vs. non-industry trials), and publication year (Table S3, Appendix S1 and S13).

Clinical trials in patients with type 2 diabetes have reported significantly greater glycemic control with once-weekly subcutaneous exenatide (S-EXE [QW]) compared with the twice-daily formulation (S-EXE [BID]) [36, 37]; thus, we categorized these as distinct treatment arms in this network meta-analysis. The primary network meta-analysis aggregated different doses and formulations of the same medication (e.g., once-daily subcutaneous liraglutide [S-LIR (QD)] at 1.8 mg and 3.0 mg; S-SEM (QW) at 1.0 mg and 2.0 mg) into a single treatment node, following the methodology of a previous network meta-analysis for individuals with obesity [38]. This approach was prespecified to preserve network connectivity and ensure statistical stability, as several dose-specific regimens were informed by a limited number of trials and would otherwise have led to sparse comparisons that could compromise the strength of the model. This strategy enables a more reliable estimation of the overall efficacy of each molecule by ensuring network stability, despite limiting the ability to distinguish dose-dependent effects in the primary network. However, we performed the following five sensitivity analyses for the primary outcome and all-cause discontinuation to verify the consistency and robustness of our findings, thereby addressing these limitations and considering clinical heterogeneity that originates from differences in clinical diagnoses, background antipsychotics, and the potential impact of bias: (1) a separate dose- and formulation-specific network meta-analysis; (2) inclusion of only RCTs where participants received clozapine and/or olanzapine; (3) exclusion of RCTs involving patients with first-episode psychosis; (4) exclusion of RCTs in which over 50% of the participants had bipolar disorder; and (5) exclusion of the study assessed to have a high overall risk of bias [39]. Finally, the findings were integrated into the Confidence in Network Meta-Analysis application—an adaptation of the Grading of Recommendations Assessment, Development, and Evaluation approach—to assess the credibility of each network meta-analysis [40–42].

## Results

### Study characteristics

Figure S1 presents the literature search flowchart and a detailed explanation of the process. A total of 504 articles were identified, of which 120 duplicates were excluded. After screening titles and abstracts, 369 articles were excluded. Further, six articles conducting post hoc analyses were excluded. The previous reviews identified no additional studies. Finally, the meta-analysis included nine trials (*n* = 595, average age = 37.8 years, 55.3% female, 83.9% schizophrenia spectrum disorders) [16–18, 39, 43–47]. The active-treatment arms included S-EXE (QW) at 2.0 mg, S-EXE (BID) at 10 μg, S-LIR (QD) at 1.8–3.0 mg, and S-SEM (QW) at 1.0–2.0 mg. Of the included studies, eight used a placebo control, whereas the remaining study utilized a non-placebo control [39]. Both were collectively defined as the control arm in this network meta-analysis. Table 1 summarizes the characteristics of the included studies. Three of the nine studies were non-industry sponsored [16, 39, 43]. The median duration of illness was 26 weeks (range: 12 − 40 weeks). One study included patients with schizophrenia spectrum disorders and bipolar disorder [46], one study involved only patients with bipolar disorder [45], and seven studies included patients with schizophrenia spectrum disorders. The McElroy study administered various psychotropic drugs, including mood stabilizers, antidepressants, and/or antipsychotics, to patients with bipolar disorder [45], whereas the remaining eight studies included patients who received antipsychotic treatment. The overall risk of bias of one study that did not employ a placebo was rated as “high” [39]; however, the remaining studies, all of which were DBRPCTs, were rated as “low.” (Table S4). The examination of study characteristics across different trials reported no evidence of transitivity violations (Table S3).

**Table 1:** Characteristics of randomized, controlled trials included in the network meta-analysis.

Study name	Sponsorship	Country	Duration	Diagnosis	T2DM (%)	Total n	PD med	Anti-DM med use (%)	Mean age (years)	Female (%)	GLP-1 RAs injection
Ganeshalingam [17]	Industry	Denmark	30 weeks	SZ (ICD-10) Prediabetes† and BMI ≥ 27	0.00	154	Stable receiving SGA (CLO 26.8%, OLA 14.3%) for ≥ 6 months	Yes (MET: 7.14)	38.3 ± 10.7	56.5	Once weekly S-SEM 1.0 mg (flexible)
Ishøy [43]	Academia	Denmark	3 months	SA or SZ (ICD-10) BMI ≥ 30	0.00	45	Stable receiving APD including CLO and OLA for ≥ 3 months	No	35.8 ± 10.3	53.3	Once weekly S-EXE 2.0 mg (fixed)
Larsen [44]	Industry	Denmark	16 weeks	SZ or PP (DSM-IV-TR or ICD-10) Prediabetes‡ and BMI ≥ 27	0.00	103	Stable receiving CLO or OLA for ≥ 6 months	No	42.5 ± 10.5	41.7	Once daily S-LIR 1.8 mg (flexible)
McElroy [45]	Industry	USA	40 weeks	BD I or II (DSM-IV-TR) BMI > 30 or BMI ≥ 27 and ≥ 1 related comorbidity including DM§	6.67¶	60	Stable receiving PD for ≥ 3 months	Yes (6.67)	42.7 ± 11.6	80.0	Once daily S-LIR 3.0 mg (flexible)
Patino [46]	Industry	USA	16 weeks	BD (66.7%), SA or SZ (DSM-IV-TR) BMI ≥ 25 or ≥ 7% weight gain while taking OLA	0.00	54	Stable receiving OLA for ≥ 4 weeks	No	41.2 ± 11.0	66.7	Twice daily S-EXE 10 μg (flexible)
Sass [18]	Industry	Denmark	26 weeks	SA, SZ or PP (DSM-V or ICD-10R) Dysglycemia†† while taking CLO or OLA	4.11	73	Stable receiving CLO or OLA for ≥ 60 months	No	35.0 ± 11.9	65.8	Once weekly S-SEM 1.0 mg (flexible)
Siskind [39]‡‡	Academia	Australia	24 weeks	SA or SZ (clinical diagnosis) BMI: 30 − 45	17.9	28	Stable receiving CLO for > 18 weeks	Yes (NR)	18 − 64 years	35.7	Once weekly S-EXE 2.0 mg (fixed)
Siskind [16]	Academia	Australia	36 weeks	SA or SZ (DSM-IV) BMI: 26 − 40 Weight loss < 5% despite ≥ 16 weeks on MET§§	0.00	31	Stable receiving CLO for ≥ 18 weeks	Yes (MET: 67.7)	38.9 ± 9.30	32.3	Once weekly S-SEM 2.0 mg (fixed)
Whicher [47]	Industry	UK	6 months	SA or SZ (ICD-10), or FEP BMI ≥ 30 or BMI ≥ 27 and ≥ 1 related comorbidity including dysglycemia¶¶	8.51	47	Stable receiving APD (CLO 19.1%, OLA 4.26%) for ≥ 1 months	No	44.0 ± 11.0	48.9	Once daily S-LIR 3.0 mg (flexible)

†HbA1C level between 5.7% and 6.4% of total hemoglobin, equivalent to 39-47 mmol/mol [to convert HbA1C from percentage of total hemoglobin to mmol/mol, use the following formula: (HbA1C % − 2.152)/0.09148].

‡Elevated fasting plasma glucose level of 110 to 125 mg/dL (to convert to mmol/L, multiply by 0.0555), elevated glycated hemoglobin level of 6.1% to 6.4% (to convert to mmol/mol, subtract 2.15% and multiply by 10.929), and/or impaired glucose tolerance with a 2-h plasma glucose level of at least 140 mg/dL during a 75-g oral glucose tolerance test.

§Hypertension, type 2 diabetes, or dyslipidemia.

¶The proportion of participants on diabetes medication.

††Either type 2 diabetes (established clinical diagnosis or HbA1c level ≥6.5% [≥48 mmol/mol]; upper limit for type 2 diabetes, 7.4% [57 mmol/mol]); or high-risk HbA1c (>5.4% [35 mmol/mol] and <6.4% [47 mmol/mol]).

‡‡This is a randomized, controlled, open-label, trial. This study did not use a placebo.

§§Prediabetes accounted for 29.0% of the participants.

¶¶Dysglycemia (prediabetes or type 2 diabetes), hypertension, dyslipidemia or obstructive sleep apnea.

*Anti-DM med* antidiabetic medication, *APD* antipsychotic drug, *BD* bipolar disorder, *BMI* body mass index, *CLO* clozapine, *DSM(-TR)* diagnostic and statistical manual of mental disorders(-text revision), *FEP* first episode psychosis, *GLP-1 RAs* glucagon-like peptide-1 receptor agonists, *ICD* international classification of diseases, *MET* metformin, n: number of individuals, *OLA* olanzapine, *PD (med)* psychotropic drug (medication), *PP* paranoid psychosis, *SA* schizoaffective disorder, *S-EXE* subcutaneous exenatide, *SGA* second generation antipsychotic, *S-LIR* subcutaneous liraglutide, *S-SEM* subcutaneous semaglutide, *SZ* schizophrenia, *(T2)DM* (type 2) diabetes mellitus, *UK* United Kingdom, *USA* United States of America.

### Network meta-analysis results

Appendix S1 provides a visual representation of the network for the primary outcome. All active treatments were directly compared only with the control, and head-to-head trials were not available; thus, the resulting star-shaped network implies that all comparisons and rankings among active agents were based exclusively on indirect evidence via the common comparator. Appendices S1–S24 detail the results of the network meta-analysis, including SUCRA rankings, heterogeneity and inconsistency assessments, and contribution matrix for each outcome.

### Primary outcome

**Fig. 1:**
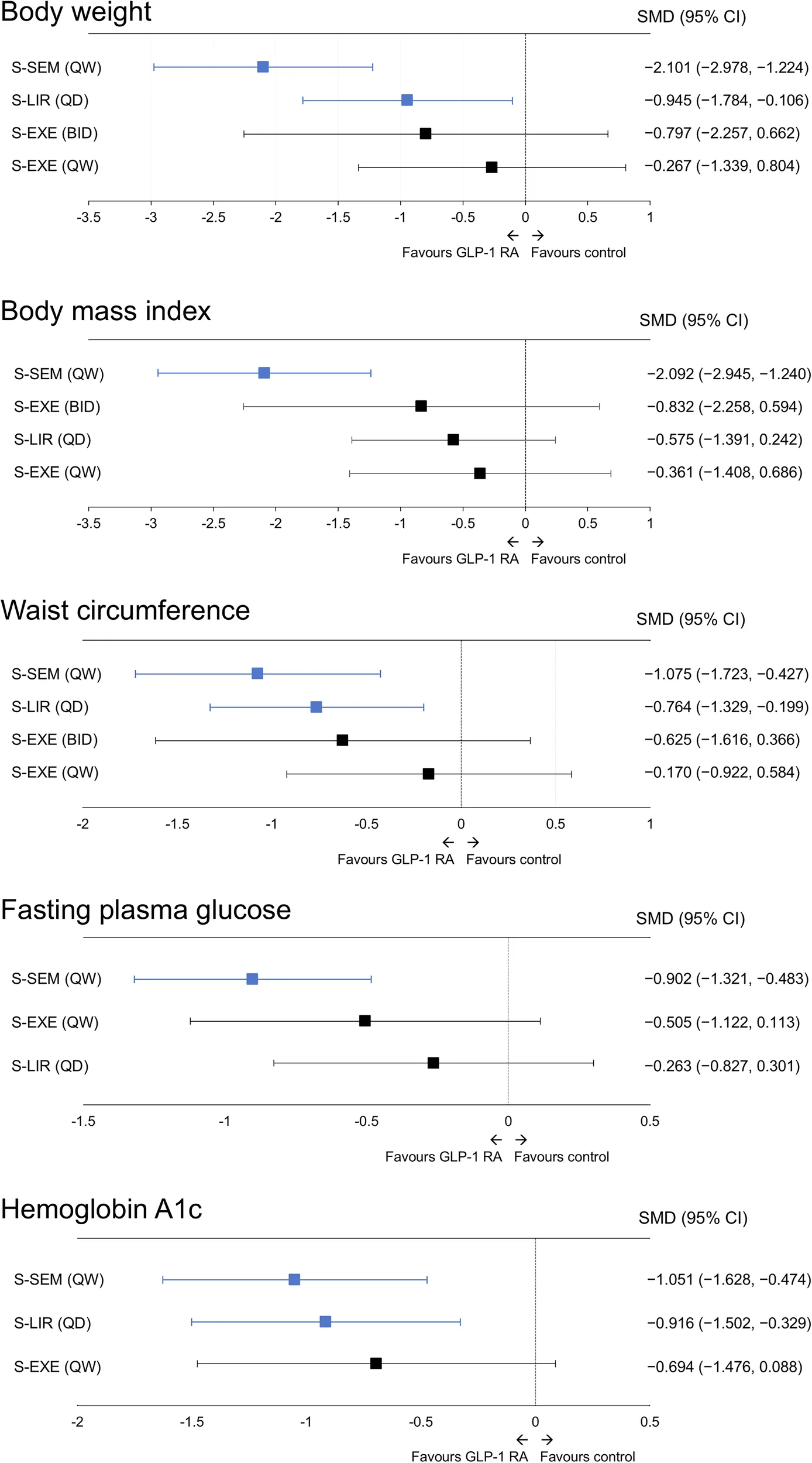
Forest plot. 95% CI: 95% confidence interval; GLP-1 RA: glucagon-like peptide-1 receptor agonist, S-EXE (BID or QW): subcutaneous exenatide (BID or QW), S-LIR (QD): subcutaneous liraglutide (QD), S-SEM (QW): subcutaneous semaglutide (QW), SMD: standardized mean difference. The blue line represents the superiority of the active treatment over the control. The black line indicates the similarity of the active treatment to the control.

S-LIR (QD) and S-SEM (QW), but not S-EXE (BID) and S-EXE (QW), were significantly associated with body weight reductions compared with control, with SMDs (95% CIs) of −0.945 ( − 1.784 to −0.106) and −2.101 ( − 2.978 to −1.224), respectively (Fig. 1 and Table 2). S-SEM (QW) was superior to S-EXE (QW) for this outcome (Table 2). A 3.0 kg change in body weight corresponds to an SMD of 0.40 (Appendix S1); thus, these values translate to MDs (95% CIs) of −7.09 kg ( − 13.38 to −0.80) for S-LIR (QD) and −15.76 kg ( − 22.34 to −9.18) for S-SEM (QW). S-SEM (QW) demonstrated the highest SUCRA values regarding outcome (Appendix S1).

**Table 2:** Metabolic outcomes.

**Body weight**				
S-EXE (BID)	-0.530 (-2.341, 1.281)	0.148 (-1.536, 1.831)	1.304 (-0.399, 3.007)	-0.797 (-2.257, 0.662)
	S-EXE (QW)	0.677 (-0.684, 2.039)	**1.834 (0.449, 3.219)**	-0.267 (-1.339, 0.804)
		S-LIR (QD)	1.156 (-0.058, 2.370)	**-0.945 (-1.784, -0.106)**
			S-SEM (QW)	**-2.101 (-2.978, -1.224)**
				Control
**Body mass index**				
S-EXE (BID)	-0.471 (-2.240, 1.298)	-0.258 (-1.900, 1.385)	1.260 (-0.401, 2.921)	-0.832 (-2.258, 0.594)
	S-EXE (QW)	0.214 (-1.114, 1.541)	**1.731 (0.381, 3.081)**	-0.361 (-1.408, 0.686)
		S-LIR (QD)	**1.518 (0.337, 2.698)**	-0.575 (-1.391, 0.242)
			S-SEM (QW)	**-2.092 (-2.945, -1.240)**
				Control
**Waist circumference**				
S-EXE (BID)	-0.456 (-1.700, 0.789)	0.139 (-1.001, 1.279)	0.450 (-0.734, 1.634)	-0.625 (-1.616, 0.366)
	S-EXE (QW)	0.595 (-0.347, 1.536)	0.906 (-0.088, 1.899)	-0.170 (-0.922, 0.584)
		S-LIR (QD)	0.311 (-0.549, 1.171)	**-0.764 (-1.329, -0.199)**
			S-SEM (QW)	**-1.075 (-1.723, -0.427)**
				Control
**Fasting plasma glucose**				
S-EXE (QW)	-0.242 (-1.078, 0.594)	0.397 (-0.349, 1.143)	-0.505 (-1.122, 0.113)	
	S-LIR (QD)	0.639 (-0.063, 1.342)	-0.263 (-0.827, 0.301)	
		S-SEM (QW)	**-0.902 (-1.321, -0.483)**	
			Control	
**Hemoglobin A1c**				
S-EXE (QW)	0.222 (-0.756, 1.199)	0.357 (-0.615, 1.329)	-0.694 (-1.476, 0.088)	
	S-LIR (QD)	0.135 (-0.688, 0.958)	**-0.916 (-1.502, -0.329)**	
		S-SEM (QW)	**-1.051 (-1.628, -0.474)**	
			Control	

*S-EXE (BID or QW)* subcutaneous exenatide (twice daily or once weekly), *S-LIR (QD)* subcutaneous liraglutide (once daily), *S-SEM (QW)* subcutaneous semaglutide (once weekly).

Comparisons should be read from left to right, with the estimate in each cell corresponding to the comparison between the column-defining and row-defining treatments. Each cell provides the standardized mean difference (SMD) with a 95% confidence interval for the corresponding comparison. Cells in bold indicate statistically significant results.

SMDs < 0 favor the row-defining treatment.

Network meta-regression analyses revealed no potential confounding factors influencing body weight effect sizes (Appendix S1).

A separate dose- and formulation-specific network meta-analysis, conducted as a sensitivity analysis, revealed that S-LIR (1.8 mg, QD), S-SEM (1.0 mg, QW), and S-SEM (2.0 mg, QW) were significantly associated with body weight reductions compared with control (Appendix S1). S-SEM (2.0 mg, QW) was superior to all other active comparators in terms of this outcome (Appendix S1). The sensitivity analysis excluding the study with a high overall risk of bias yielded results consistent with the primary analysis; however, the remaining three sensitivity analyses revealed that only S-SEM (QW) was significantly related to decreased body weight compared with the control (Appendix S1).

### Outcomes related to metabolism and death

S-LIR (QD) was associated with waist circumference (Appendix 3) and blood HbA1c level (Appendix S5) reductions compared with control (Fig. 1 and Table 2). S-SEM (QW) was associated with the decreases in BMI (Appendix S2), waist circumference (Appendix S3), fasting plasma glucose (Appendix S4), and HbA1c levels (Appendix S5) compared with control (Fig. 1 and Table 2). No significant differences in these outcomes were observed between S-EXE (BID) or S-EXE (QW) and the control (Fig. 1 and Table 2). Furthermore, none of the GLP-1 RAs significantly differed from the control regarding lipid-related outcomes (Appendices S6–S9), blood pressure (Appendices S10–S11), or mortality (Appendix S12). The supplementary information presented values converted from SMD to MD (Appendices S2–S11).

### Outcomes related to acceptability, tolerability, and safety

S-LIR (QD) was associated with a lower incidence of SAEs (Appendix S15) and higher nausea (Appendix S19), vomiting (Appendix S20), and constipation (Appendix S21) compared with control (Table 3). S-SEM (QW) was associated with higher incidences of nausea (Appendix S19), vomiting (Appendix S20), and constipation (Appendix S21) compared with control (Table 3). Appendices S15, S19, S20, and S21 present the RDs and NNTHs for these outcomes, where network meta-analysis revealed significant between-group differences based on ORs. No significant differences were observed between S-EXE (BID) or S-EXE (QW) and the control for any of these outcomes (Table 3). Furthermore, none of the individual GLP-1 RAs significantly differed from the control regarding treatment discontinuation (Table 3, Appendices S13–S14), the incidence of headache (Appendix S17), dizziness (Appendix S18), or diarrhea (Appendix S22). Similarly, no significant differences in the incidence of psychiatric hospitalization during the trial (Table 3, Appendix S23) or mean change from baseline to endpoint in overall schizophrenia symptom scores as measured by standardized rating scales (Table 3, Appendix S24) were observed between any GLP-1 RA and the control.

**Table 3:** Acceptability and safety outcomes.

**All cause discontinuation**				
S-EXE (BID)	1.053 (0.060, 18.464)	1.018 (0.103, 10.091)	2.697 (0.272, 26.744)	1.429 (0.210, 9.707)
	S-EXE (QW)	0.967 (0.081, 11.482)	2.562 (0.216, 30.432)	1.357 (0.161, 11.408)
		S-LIR (QD)	2.650 (0.445, 15.776)	1.404 (0.398, 4.955)
			S-SEM (QW)	0.530 (0.150, 1.870)
				Control
**Serious adverse events**				
S-EXE (BID)	6.152 (0.289, 130.830)	7.618 (0.549, 105.626)	3.789 (0.296, 48.419)	2.636 (0.224, 30.969)
	S-EXE (QW)	1.238 (0.163, 9.432)	0.616 (0.090, 4.216)	0.429 (0.070, 2.620)
		S-LIR (QD)	0.497 (0.161, 1.533)	**0.346 (0.138, 0.868)**†
			S-SEM (QW)	0.696 (0.363, 1.333)
				Control
**Nausea**				
S-EXE (QW)	0.654 (0.143, 2.993)	1.061 (0.233, 4.824)	1.969 (0.485, 7.991)	
	S-LIR (QD)	1.623 (0.710, 3.711)	**3.012 (1.664, 5.453)**‡	
		S-SEM (QW)	**1.856 (1.043, 3.301)**§	
			Control	
**Vomiting**				
S-EXE (QW)	0.420 (0.022, 8.014)	0.303 (0.016, 5.670)	0.955 (0.056, 16.265)	
	S-LIR (QD)	0.721 (0.240, 2.165)	**2.275 (1.010, 5.127)**¶	
		S-SEM (QW)	**3.155 (1.505, 6.615)**††	
			Control	
**Constipation**				
S-LIR (QD)	0.797 (0.244, 2.598)	**3.136 (1.426, 6.897)**‡‡		
	S-SEM (QW)	**3.937 (1.632, 9.499)**§§		
		Control		
**Psychiatric hospitalization**				
S-EXE (BID)	0.164 (0.003, 10.284)	4.140 (0.187, 91.423)	1.996 (0.109, 36.401)	1.261 (0.075, 21.266)
	S-EXE (QW)	25.227 (0.953, 667.874)	12.163 (0.550, 268.943)	7.683 (0.374, 157.906)
		S-LIR (QD)	0.482 (0.115, 2.013)	0.305 (0.086, 1.077)
			S-SEM (QW)	0.632 (0.324, 1.233)
				Control
**Overall symptoms**				
S-EXE (QW)	-0.514 (-1.354, 0.326)	0.124 (-0.412, 0.659)	0.093 (-0.383, 0.569)	
	S-LIR (QD)	0.638 (-0.096, 1.372)	0.607 (-0.085, 1.300)	
		S-SEM (QW)	-0.030 (-0.275, 0.214)	
			Control	

*S-EXE (BID or QW)* subcutaneous exenatide (twice daily or once weekly), *S-LIR (QD)* subcutaneous liraglutide (once daily), *S-SEM (QW)* subcutaneous semaglutide (once weekly)

Comparisons should be read from left to right, with the estimate in each cell corresponding to the comparison between the column-defining and row-defining treatments. For all outcomes except overall symptoms, results are presented as odds ratios (ORs) with 95% confidence intervals (CIs). For overall symptoms, standardized mean differences (SMDs) with 95% CIs are reported. Cells in bold indicate statistically significant results.

SMDs < 0 favor the row-defining treatment.

ORs < 1 favor the row-defining treatment.

Risk differences (RDs) and numbers needed to treat to harm (NNTHs) were calculated only when network meta-analysis revealed significant between-group differences based on ORs.

†RD: -0.095 (-0.175, -0.016), NNTH: -10.5 (-62.5, -5.7)

‡RD: 0.263 (0.129, 0.398), NNTH: 3.8 (2.5, 7.8)

§RD: 0.133 (0.029, 0.237), NNTH: 7.5 (4.2, 34.5)

¶RD: not significant

††RD: 0.171 (0.010, 0.332), NNTH: 5.8 (3.0, 100.0)

‡‡RD: 0.217 (0.096, 0.337), NNTH: 4.6 (3.0, 10.4)

§§RD: 0.182 (0.098, 0.266), NNTH: 5.5 (3.8, 10.2)

Network meta-regression analyses revealed no potential confounders influencing the effect sizes for all-cause discontinuation (Appendix S13). Furthermore, all sensitivity analyses for this outcome generated results consistent with the primary analysis (Appendix S13).

### Heterogeneity, inconsistency, and network meta-analysis results were graded using the CINeMA application

Appendices S1–S24 present the results for heterogeneity and inconsistency for all outcomes. Global heterogeneity was assessed as “moderate-to-high” or “high” in 50.0% of the outcomes, including the primary outcome and all-cause discontinuation. None of the sensitivity analyses for these outcomes reduced the substantial global heterogeneity. We reported considerable local heterogeneity for 41.7% of the outcomes in the specific comparisons and detected significant global inconsistency in 29.2% of the outcomes, including the primary outcome. Local inconsistency could not be assessed because the networks for these outcomes contained no closed loops from head-to-head comparisons. The majority of comparisons were assessed as having “some concerns” regarding within-study bias. According to fewer than 10 studies, funnel plots are considered unreliable, and none of the comparisons included 10 or more studies; thus, publication bias was not evaluated [20]. Therefore, all comparisons for publication bias were assessed as “some concerns.” Comparisons based only on indirect evidence were downgraded by one level. Consequently, the majority of the evidence comparing active treatments with the control (including direct evidence) was assessed as having “moderate” overall confidence, whereas most comparisons between active treatments (i.e., indirect evidence) were evaluated as having “very low” confidence.

## Discussion

Compared with previous meta-analyses [9, 10], this current network meta-analysis demonstrates twofold strengths. First, it includes trials of S-SEM (QW), the recommended first-line treatment for individuals with obesity [6, 7, 11–14]. Second, it comprehensively evaluates acceptability, tolerability, and safety outcomes. Among the three GLP-1 RAs assessed, S-SEM (QW) produced the greatest body weight reduction in individuals with obesity comorbid with mental illness and was the only agent demonstrating consistent weight loss across all sensitivity analyses. Moreover, S-SEM (QW) significantly reduced BMI, waist circumference, fasting plasma glucose, and HbA1c levels. S-SEM (QW) increases the risk of nausea, vomiting, and constipation; however, the trend toward reducing all-cause discontinuation, SAE incidence, and psychiatric hospitalization may indicate a well-accepted treatment by this population, thereby suggesting potential benefits in preventing the exacerbation of both physical and psychiatric illnesses. Moreover, S-LIR (QD) reduced body weight, waist circumference, and HbA1c levels in this population. S-LIR (QD) increased the risk of nausea, vomiting, and constipation; however, S-LIR (QD) decreased the risk of SAEs and its trend toward reducing psychiatric hospitalization. S-EXE (BID) and S-EXE (QW) were associated with no risk of individual adverse events, but they demonstrated no efficacy for metabolism-related outcomes. Therefore, S-SEM (QW) might be considered a preferable treatment option for individuals with obesity comorbid with mental illness. Compared with S-LIR (QD), S-SEM (QW) may have a larger effect size for decreasing body weight in not only the general population with obesity but also in those with obesity comorbid with mental illness [13, 15]. However, comparative ranking remains unclear due to sparse networks, substantial heterogeneity, and predominantly indirect evidence.

A separate dose- and formulation-specific network meta-analysis revealed that S-SEM (QW), but not S-LIR (QD), increased the effect size for body weight dose-dependently. Clinical trials in patients with type 2 diabetes have reported significantly greater glycemic control with S-EXE (QW) compared with S-EXE (BID) [36, 37]; however, our network meta-analysis revealed that S-EXE (BID) appeared to yield a larger effect size than S-EXE (QW). These results should be interpreted as exploratory, along with the aforementioned limitations, as the number of studies in each treatment arm was small.

Beyond metabolic benefits, the weight loss observed with GLP-1 RAs may carry important translational implications for psychiatric management. First, weight gain is a primary driver of non-adherence to antipsychotic medication; thus, mitigating this side effect could indirectly improve long-term treatment persistence [48, 49]. Second, reductions in adipose tissue may decrease systemic inflammation, potentially attenuating neuroinflammation. This is one of the key factors in cognitive impairment across both disorders, including the negative symptoms specific to schizophrenia spectrum disorders and the mood symptoms in bipolar disorder [50–53]. Furthermore, weight loss could improve sleep quality by reducing the risk of obstructive sleep apnea, thereby improving daytime energy and overall psychosocial functioning [54]. Future trials should move beyond simple anthropometric measurements and incorporate biomarkers of inflammation, objective sleep metrics, and standardized assessments of cognition and negative symptoms to validate the intricate mechanistic association between metabolic and psychiatric pathways.

The implementation of GLP-1 RAs in psychiatric settings may warrant a multidisciplinary approach tailored to the unique needs of people with severe mental illness (SMI). Coordination with endocrinology is crucial to harmonize metabolic monitoring with psychiatric care. A “low and slow” titration strategy is recommended to manage gastrointestinal adverse events, including nausea, vomiting, and constipation [55]. This approach is particularly important for patients with SMI, as they may experience challenges in communicating their discomfort, and can help improve overall tolerance.

The GLP-1 RAs investigated in our study are self-injected subcutaneously. This delivery method ensures superior bioavailability by bypassing the first-pass effect, despite injection-related discomfort. Long-term treatment is essential for diabetes and obesity, and adults in the general population frequently prefer the flexibility of oral formulations [56]. However, the clinical landscape considerably differs for individuals with SMI, of whom adherence is frequently suboptimal. Consequently, long-acting injectable (LAI) antipsychotics are established therapeutic options for schizophrenia spectrum and bipolar disorders [1, 35, 48, 57]. Hence, injectable formulations may provide a strategic advantage for individuals with SMI. However, providing daily injection support could strain medical resources and may lack clinical feasibility in resource-limited community settings. Conversely, a weekly formulation reduces the frequency of administration, thereby minimizing the workload for healthcare providers and potentially increasing acceptability among patients already accustomed to LAI antipsychotics. Synchronizing these treatments within Assertive Community Treatment frameworks could simplify complex medication regimens and improve long-term treatment persistence in this vulnerable population.

Interestingly, S-LIR (QD) was associated with a lower SAE incidence compared with the control. Further, S-SEM (QW) demonstrated a trend toward reduced SAE incidence. Specifically, the Larsen study (16-week duration) reported exacerbations of psychiatric illness and voluntary psychiatric hospitalizations as the most common SAEs. These events were infrequent with S-LIR (QD) (6.0%) compared with placebo (17.7%) [44]. In our network meta-analysis, S-LIR (QD) and S-SEM (QW) showed a trend toward reduced psychiatric hospitalization incidences. These benefits could enhance adherence to psychotropic medications and improve psychiatric stability, driven by weight loss and better glycemic control.

No significant differences in overall schizophrenia symptom severity were observed between any GLP-1 RAs and control groups. Participants in the studies contributing to this outcome were diagnosed exclusively with schizophrenia spectrum disorders, and their symptoms were assessed utilizing standardized clinical scales (Table S2). However, these non-significant findings warrant cautious interpretation. Due to the limited number of studies (k = 6), the statistical power was likely insufficient to detect subtle clinical differences, and the wide CIs reflect constrained precision. Furthermore, all included studies involved participants who were clinically stable at baseline; thus, a “floor effect” may have limited the potential for further measurable symptomatic improvement. The insufficient available data to rigorously evaluate significant differences in overall schizophrenia symptom improvement between the active and control groups warrants cautious interpretation of the findings.

Sensitivity analysis focusing on olanzapine and clozapine suggests that S-SEM (QW) may be particularly effective in mitigating weight gain induced by these high-risk antipsychotics [48, 58–62]. Conversely, whether the efficacy of GLP-1 receptor agonists significantly differs when used alongside psychotropic drugs with lower weight-gain propensities remains unclear. Metabolic risks vary widely across both psychiatric diagnostic groups and individual psychotropic agents [48, 58–62]; thus, future research should clarify which GLP-1 receptor agonists and specific treatment regimens provide the greatest efficacy for metabolic impairments induced by particular medications within distinct patient populations.

Our study has several limitations. First, our meta-analysis included a small number of studies and participants. Second, global heterogeneity reached a “moderate-to-high” or “high” level in some outcomes, including body weight and all-cause discontinuation. A substantial local heterogeneity was observed in half of the outcomes across several specific comparisons. Network meta-regression and sensitivity analyses were performed for body weight and all-cause discontinuation; however, no significant confounding factors were detected. Moreover, local inconsistency could not be assessed because head-to-head comparisons were unavailable for all outcomes. Third, the trials included in our network meta-analysis did not report SAEs, including hypoglycemia, acute pancreatitis, or ileus; thus, these adverse events were not assessed. Fourth, our study did not address certain practical aspects relevant to clinical decision-making, including integration with other pharmacological or nonpharmacological interventions and cost-effectiveness considerations. Fifth, the baseline use of concomitant glucose-lowering therapies, such as metformin, may have influenced our findings. Table 1 shows that no study has exclusively enrolled participants receiving a combination of metformin and GLP-1 RAs. Therefore, further primary studies focusing on this specific drug combination are warranted to clarify these interactions. Finally, a clearer understanding of the similarities and differences between GLP-1 RAs and other medications, including metformin, warrants adequately powered, randomized, head-to-head trials to enable direct comparisons.

In conclusion, S-SEM (QW) may be the preferred treatment option, given its largest estimated effects on body weight compared with control. However, the comparative ranking remains uncertain due to limited network connectivity, substantial heterogeneity, and predominance of indirect evidence. This warrants cautious interpretation of the findings as preliminary evidence.

## Supplementary information

Supplementary material

## Data Availability

The datasets generated during and/or analyzed during the current study are available from the corresponding author on reasonable request.
