# Supplementary material for: Glucagon-like peptide-1 receptor agonists for weight management in mental illness: a network meta-analysis
Source: Transl Psychiatry. 2026 Jul 8;16:507. doi: 10.1038/s41398-026-04253-6 (PMC13631319; doi:10.1038/s41398-026-04253-6)
Supplement: Supplementary file 1 [file 41398_2026_4253_MOESM1_ESM.docx]

**Figure S1. PRISMA 2020 flow diagram.**

**Table S1. PRISMA for Network Meta-Analyses Checklist.**

| **Section/Topic** | **Item #** | **Checklist Item** | **Reported on Page #** |
| --- | --- | --- | --- |
| **TITLE** |  |  |  |
| Title | 1 | Identify the report as a systematic review *incorporating a network meta-analysis (or related form of meta-analysis).* | 1 |
| **ABSTRACT** |  |  |  |
| Structured summary | 2 | Provide a structured summary including, as applicable:  **Background:** main objectives  **Methods:** data sources; study eligibility criteria, participants, and interventions; study appraisal; and *synthesis methods, such as network meta-analysis.*  **Results:** number of studies and participants identified; summary estimates with corresponding confidence/credible intervals; *treatment rankings may also be discussed. Authors may choose to summarize pairwise comparisons against a chosen treatment included in their analyses for brevity.*  **Discussion/Conclusions:** limitations; conclusions and implications of findings.  **Other:** primary source of funding; systematic review registration number with registry name. | 3- |
| **INTRODUCTION** |  |  |  |
| Rationale | 3 | Describe the rationale for the review in the context of what is already known*, including mention of why a network meta-analysis has been conducted.* | 5- |
| Objectives | 4 | Provide an explicit statement of questions being addressed, with reference to participants, interventions, comparisons, outcomes, and study design (PICOS). | 5- |
| **METHODS** |  |  |  |
| Protocol and registration | 5 | Indicate whether a review protocol exists and if and where it can be accessed (e.g., Web address); and, if available, provide registration information, including registration number. | 6- |
| Eligibility criteria | 6 | Specify study characteristics (e.g., PICOS, length of follow-up) and report characteristics (e.g., years considered, language, publication status) used as criteria for eligibility, giving rationale. *Clearly describe eligible treatments included in the treatment network, and note whether any have been clustered or merged into the same node (with justification).* | 6- |
| Information sources | 7 | Describe all information sources (e.g., databases with dates of coverage, contact with study authors to identify additional studies) in the search and date last searched. | 6- |
| Search | 8 | Present full electronic search strategy for at least one database, including any limits used, such that it could be repeated. | 6- |
| Study selection | 9 | State the process for selecting studies (i.e., screening, eligibility, included in systematic review, and, if applicable, included in the meta-analysis). | 6- |
| Data collection process | 10 | Describe method of data extraction from reports (e.g., piloted forms, independently, in duplicate) and any processes for obtaining and confirming data from investigators. | 6- |
| Data items | 11 | List and define all variables for which data were sought (e.g., PICOS, funding sources) and any assumptions and simplifications made. | 6- |
| **Geometry of the network** | **S1** | Describe methods used to explore the geometry of the treatment network under study and potential biases related to it. This should include how the evidence base has been graphically summarized for presentation, and what characteristics were compiled and used to describe the evidence base to readers. | 6- |
| Risk of bias within individual studies | 12 | Describe methods used for assessing risk of bias of individual studies (including specification of whether this was done at the study or outcome level), and how this information is to be used in any data synthesis. | 6- |
| Summary measures | 13 | State the principal summary measures (e.g., risk ratio, difference in means). *Also describe the use of additional summary measures assessed, such as treatment rankings and surface under the cumulative ranking curve (SUCRA)* values, as well as modified approaches used to present summary findings from meta-analyses.* | 6- |
| Planned methods of analysis | 14 | Describe the methods of handling data and combining results of studies for each network meta-analysis. This should include, but not be limited to:   - *Handling of multi-arm trials;* - *Selection of variance structure;* - *Selection of prior distributions in Bayesian analyses; and* - *Assessment of model fit.* | 6- |
| **Assessment of Inconsistency** | **S2** | Describe the statistical methods used to evaluate the agreement of direct and indirect evidence in the treatment network(s) studied. Describe efforts taken to address its presence when found. | 6- |
| Risk of bias across studies | 15 | Specify any assessment of risk of bias that may affect the cumulative evidence (e.g., publication bias, selective reporting within studies). | 6- |
| Additional analyses | 16 | Describe methods of additional analyses if done, indicating which were pre-specified. This may include, but not be limited to, the following:   - Sensitivity or subgroup analyses; - Meta-regression analyses; - *Alternative formulations of the treatment network; and* - *Use of alternative prior distributions for Bayesian analyses (if applicable).* | 6- |
| **RESULTS†** |  |  |  |
| Study selection | 17 | Give numbers of studies screened, assessed for eligibility, and included in the review, with reasons for exclusions at each stage, ideally with a flow diagram. | 10- |
| **Presentation of network structure** | **S3** | Provide a network graph of the included studies to enable visualization of the geometry of the treatment network. | 10- |
| **Summary of network geometry** | **S4** | Provide a brief overview of characteristics of the treatment network. This may include commentary on the abundance of trials and randomized patients for the different interventions and pairwise comparisons in the network, gaps of evidence in the treatment network, and potential biases reflected by the network structure. | 10- |
| Study characteristics | 18 | For each study, present characteristics for which data were extracted (e.g., study size, PICOS, follow-up period) and provide the citations. | 10- |
| Risk of bias within studies | 19 | Present data on risk of bias of each study and, if available, any outcome level assessment. | 10- |
| Results of individual studies | 20 | For all outcomes considered (benefits or harms), present, for each study: 1) simple summary data for each intervention group, and 2) effect estimates and confidence intervals. *Modified approaches may be needed to deal with information from larger networks.* | 10- |
| Synthesis of results | 21 | Present results of each meta-analysis done, including confidence/credible intervals. *In larger networks, authors may focus on comparisons versus a particular comparator (e.g. placebo or standard care), with full findings presented in an appendix. League tables and forest plots may be considered to summarize pairwise comparisons.* If additional summary measures were explored (such as treatment rankings), these should also be presented. | 10- |
| **Exploration for inconsistency** | **S5** | Describe results from investigations of inconsistency. This may include such information as measures of model fit to compare consistency and inconsistency models, *P* values from statistical tests, or summary of inconsistency estimates from different parts of the treatment network. | 10- |
| Risk of bias across studies | 22 | Present results of any assessment of risk of bias across studies for the evidence base being studied. | 10- |
| Results of additional analyses | 23 | Give results of additional analyses, if done (e.g., sensitivity or subgroup analyses, meta-regression analyses*, alternative network geometries studied, alternative choice of prior distributions for Bayesian analyses,* and so forth). | 10- |
| **DISCUSSION** |  |  |  |
| Summary of evidence | 24 | Summarize the main findings, including the strength of evidence for each main outcome; consider their relevance to key groups (e.g., healthcare providers, users, and policy-makers). | 14- |
| Limitations | 25 | Discuss limitations at study and outcome level (e.g., risk of bias), and at review level (e.g., incomplete retrieval of identified research, reporting bias). *Comment on the validity of the assumptions, such as transitivity and consistency. Comment on any concerns regarding network geometry (e.g., avoidance of certain comparisons).* | 14- |
| Conclusions | 26 | Provide a general interpretation of the results in the context of other evidence, and implications for future research. | 14- |
|  |  |  |  |
| **FUNDING** |  |  | 19 |
| Funding | 27 | Describe sources of funding for the systematic review and other support (e.g., supply of data); role of funders for the systematic review. This should also include information regarding whether funding has been received from manufacturers of treatments in the network and/or whether some of the authors are content experts with professional conflicts of interest that could affect use of treatments in the network. |  |

**Table S2. Summary of data synthesis: psychiatric hospitalization and endpoint overall symptom scores**

| Study name | GLP-1 RAs | Study duration | Psychiatric diagnosis | Psychiatric hospitalization during the trial | Endpoint overall symptom scores |
| --- | --- | --- | --- | --- | --- |
| Ganeshalingam 2025 | S-SEM (1.0 mg, QW) | 30 weeks | SZ | ✓ | ✓ (PANSS-6) |
| Ishøy 2017 | S-EXE (2.0 mg, QW) | 3 months | SA or SZ | ✓ | ✓ (PANSS) |
| Larsen 2017 | S-LIR (1.8 mg, QD) | 16 weeks | SZ or PP | ✓ |  |
| McElroy 2024 | S-LIR (3.0 mg, QD) | 40 weeks | BD I or II | ✓ |  |
| Patino 2025 | S-EXE (10 μg, BID) | 16 weeks | BD (66.7%), SA or SZ | ✓ |  |
| Sass 2025 | S-SEM (1.0 mg, QW) | 26 weeks | SA, SZ or PP | ✓ | ✓ (PANSS-6) |
| Siskind 2018 | S-EXE (2.0 mg, QW) | 24 weeks | SA or SZ |  | ✓ (BPRS) |
| Siskind 2025 | S-SEM (2.0 mg, QW) | 36 weeks | SA or SZ |  | ✓ (PANSS) |
| Whicher 2021 | S-LIR (3.0 mg, QD) | 6 months | SA or SZ, or FEP |  | ✓ (BPRS) |

BD: bipolar disorder, BPRS: Brief Psychiatric Rating Scale, FEP: first episode psychosis, PANSS: Positive and Negative Syndrome Scale, PP: paranoid psychosis, SA: schizoaffective disorder, SZ: schizophrenia

**Table S3. Transitivity assessment.**

|  | Boxplot | Differences between groups were assessed using the Kruskal–Wallis test for continuous variables and the Pearson chi-squared test for binary and categorical variables. The Fisher’s exact test was applied if more than 20% of cells had an expected frequency below 5. Transitivity violation was defined using a threshold of p > 0.1. |
| --- | --- | --- |
| Proportion of females (k = 9) |  | Chi-squared with ties = 1.89 (df = 3), p = 0.596 |
| Mean age (k = 8) |  | Chi-squared with ties = 5.89 (df = 3), p = 0.117 |
| Body weight at baseline (k = 9) |  | Chi-squared with ties = 3.49 (df = 3), p = 0.322 |
| Baseline glycemic status (studies involving patients without prediabetes or dysglycemia vs. those with these conditions) (k = 9) |  | Chi-squared with ties = 2.50 (df = 3), p = 0.100 |
| Study duration (k = 9) |  | Chi-squared with ties = 4.09 (df = 3), p = 0.252 |
| Total participants (k = 9) |  | Chi-squared with ties = 3.20 (df = 3), p = 0.362 |
| Funding source (industry-sponsored vs. non-industry trials) (k = 9) |  | Chi-squared with ties = 6.00 (df = 3), p = 0.112 |
| Publication year (k = 9) |  | Chi-squared with ties = 6.89 (df = 3), p = 0.101 |

**Table S4. Risk of bias summary**

|  | Randomization process | Deviation from intended intervention | Missing outcome data | Measurement of the outcome | Selection of the reported result | Overall risk of bias |
| --- | --- | --- | --- | --- | --- | --- |
| Ganeshalingam 2025 | Low risk | Low risk | Low risk | Low risk | Low risk | Low risk |
| Ishøy 2017 | Low risk | Low risk | Low risk | Low risk | Low risk | Low risk |
| Larsen 2017 | Low risk | Low risk | Low risk | Low risk | Low risk | Low risk |
| McElroy 2024 | Low risk | Low risk | Low risk | Low risk | Low risk | Low risk |
| Patino 2025 | Low risk | Low risk | Low risk | Low risk | Low risk | Low risk |
| Sass 2025 | Low risk | Low risk | Low risk | Low risk | Low risk | Low risk |
| Siskind 2018 | Low risk | High risk | Low risk | High risk | Low risk | High risk |
| Siskind 2025 | Low risk | Low risk | Low risk | Low risk | Low risk | Low risk |
| Whicher 2021 | Low risk | Low risk | Low risk | Low risk | Low risk | Low risk |

**Appendix S1. Body weight**

9 studies, 567 participants

Node size by sample size

Node color by risk of bias

Green: low overall risk of bias

Red: high overall risk of bias

Edge width by number of studies

Edge color by: majority of indirectness

S-EXE: subcutaneous exenatide

S-LIR, subcutaneous liraglutide

S-SEM: subcutaneous semaglutide

CON: control

| Abbreviation | English meaning | Latin origin |
| --- | --- | --- |
| QW | once weekly | quaque week |
| QD | once daily (every day) | quaque die |
| BID | twice daily (two times a day) | bis in die |

**League table (SMD with 95% CI)**

| S-EXE (BID) | -0.530 (-2.341, 1.281) | 0.148 (-1.536, 1.831) | 1.304 (-0.399, 3.007) | -0.797 (-2.257, 0.662) |
| --- | --- | --- | --- | --- |
|  | S-EXE (QW) | 0.677 (-0.684, 2.039) | **1.834 (0.449, 3.219)** | -0.267 (-1.339, 0.804) |
|  |  | S-LIR (QD) | 1.156 (-0.058, 2.370) | **-0.945 (-1.784, -0.106)** |
|  |  |  | S-SEM (QW) | **-2.101 (-2.978, -1.224)** |
|  |  |  |  | CON |

A 3.0 kg change in body weight corresponds to an SMD of 0.40.

**SUCRA**

| S-EXE (BID) | 0.629 |
| --- | --- |
| S-EXE (QW) | 0.511 |
| S-LIR (QD) | 0.779 |
| S-SEM (QW) | 0.977 |
| CON | 0.011 |

**Global heterogeneity**

As previously suggested (Huhn 2019), the common 𝜏^2^ was compared to the empirical distributions of heterogeneity found in the meta‐analyses of pharmacological treatments for mental health outcomes, with a median of the 𝜏^2^ distribution of 0.049 and an inter‐quartile range of 0.010 to 0.242 (Rhodes 2015), and the heterogeneity was considered low when the estimated 𝜏^2^ was below the 25% quartile, moderate when between 25% and 50% of the quartile, and high when above the 50% quartile.

Huhn M, et al. Lancet 2019;394(10202):939-51

Rhodes KM, et al. J Clin Epidemiol 2015;68(1):52-60

Between study variance (𝜏^2^): 0.684 (heterogeneity assessment: high)

**Random-effects design-by-treatment interaction model**

p < 0.1 was considered as considerable global inconsistency.

χ^2^  statistic: 31.172 (5 degrees of freedom), P value: 0.000

**Local heterogeneity (I^2^) and incoherence (SIDE test)**

I^2^ ≥ 50% was considered as considerable heterogeneity.

SIDE test was not feasible because of the absence of closed loops and direct head-to-head comparisons between active drugs, which precluded a statistical evaluation of local inconsistency.

|  | I^2^ (%) |
| --- | --- |
| S-EXE (BID) vs CON | na |
| S-EXE (QW) vs CON | 86.0 |
| S-LIR (QD) vs CON | 8.3 |
| S-SEM (QW) vs CON | 90.8 |

**Contribution matrix**

| random SMD | S-EXE (BID) vs CON | S-EXE (QW) vs CON | S-LIR (QD) vs CON | S-SEM (QW) vs CON |
| --- | --- | --- | --- | --- |
| Mixed estimates |  |  |  |  |
| S-EXE (BID) vs CON | S-EXE (BID) vs CON: 100 | S-EXE (QW) vs CON: 0 | S-LIR (QD) vs CON: 0 | S-SEM (QW) vs CON: 0 |
| S-EXE (QW) vs CON | S-EXE (BID) vs CON: 0 | S-EXE (QW) vs CON: 100 | S-LIR (QD) vs CON: 0 | S-SEM (QW) vs CON: 0 |
| S-LIR (QD) vs CON | S-EXE (BID) vs CON: 0 | S-EXE (QW) vs CON: 0 | S-LIR (QD) vs CON: 100 | S-SEM (QW) vs CON: 0 |
| S-SEM (QW) vs CON | S-EXE (BID) vs CON: 0 | S-EXE (QW) vs CON: 0 | S-LIR (QD) vs CON: 0 | S-SEM (QW) vs CON: 100 |
| Indirect estimates |  |  |  |  |
| S-EXE (BID) vs S-EXE (QW) | S-EXE (BID) vs CON: 50 | S-EXE (QW) vs CON: 50 | S-LIR (QD) vs CON: 0 | S-SEM (QW) vs CON: 0 |
| S-EXE (BID) vs S-LIR (QD) | S-EXE (BID) vs CON: 50 | S-EXE (QW) vs CON: 0 | S-LIR (QD) vs CON: 50 | S-SEM (QW) vs CON: 0 |
| S-EXE (BID) vs S-SEM (QW) | S-EXE (BID) vs CON: 50 | S-EXE (QW) vs CON: 0 | S-LIR (QD) vs CON: 0 | S-SEM (QW) vs CON: 50 |
| S-EXE (QW) vs S-LIR (QD) | S-EXE (BID) vs CON: 0 | S-EXE (QW) vs CON: 50 | S-LIR (QD) vs CON: 50 | S-SEM (QW) vs CON: 0 |
| S-EXE (QW) vs S-SEM (QW) | S-EXE (BID) vs CON: 0 | S-EXE (QW) vs CON: 50 | S-LIR (QD) vs CON: 0 | S-SEM (QW) vs CON: 50 |
| S-LIR (QD) vs S-SEM (QW) | S-EXE (BID) vs CON: 0 | S-EXE (QW) vs CON: 0 | S-LIR (QD) vs CON: 50 | S-SEM (QW) vs CON: 50 |

Contribution of each comparison (%)

**Funnel plot (all drugs vs. control)**

As none of the comparisons included 10 or more studies, the assessment of publication bias was not performed.

**CINeMA confidence rating**

CINeMA is a web application that simplifies the evaluation of confidence in the findings from a network meta-analysis. CINeMA is based on a methodological framework described in the following articles, which consider the following six domains: within-study bias, reporting bias, indirectness, imprecision, heterogeneity, and incoherence. CINeMA grades the confidence in the results of each treatment comparison as high, moderate, low, or very low. If the comparison had only indirect evidence, the comparison was downgraded one level. The quality of evidence was also downgraded by one level if two or more domains were assessed as having “Some concerns.”

Nikolakopoulou A, et al., PLOS Medicine 2020 17 1-19, Papakonstantinou T, et al., Campbell Systematic Reviews 2020 16 e1080

(1) Within-study bias: Risk of bias in RCTs for the main outcomes was assessed independently using the Cochrane risk-of-bias tool for randomized trials (RoB 2). In all studies, the overall risk of bias was evaluated as “low risk.” Selected rule: Average

(2) Reporting bias: Comparison-adjusted funnel plots with less than 10 studies are not meaningful. Therefore, all comparisons were “Some concerns.”

(3) Indirectness: No indirectness was assumed. Selected rule: Average

(4) Imprecision: Standardized mean differences (SMDs) were calculated for continuous variables. To improve clinical interpretability, we provided back-transformed mean differences (MDs) alongside the SMDs. According to the methodology recommended in the Cochrane Handbook, we calculated the pooled standard deviation (SD) of the placebo groups—excluding non-placebo data—from the studies that used standard units. The SMDs derived from our network meta-analysis were then multiplied by this SD to calculate the MDs. For non active-controlled comparisons, we evaluated whether the 95% CIs excluded these SMD-converted thresholds. For active-controlled comparisons, the MID was defined as half of the placebo-controlled threshold, accounting for the smaller effect sizes typically expected between active treatments. Regarding dichotomous outcomes, the clinically meaningful threshold for non active-control comparisons was set at an OR higher or lower than 1.0. For comparisons between two active treatments, the thresholds were set at ORs of 0.80 and 1.25.

(5) Heterogeneity: We used recommendations automatically provided by CINeMA.

(6) Incoherence: SIDE test was not feasible because of the absence of closed loops and direct head-to-head comparisons between active drugs, which precluded a statistical evaluation of local inconsistency. Therefore, all indirect comparisons were “Some concerns.”

| Comparison | Number of studies | Within-study bias | Reporting bias | Indirectness | Imprecision | Heterogeneity | Incoherence | Confidence rating |
| --- | --- | --- | --- | --- | --- | --- | --- | --- |
| S-EXE (BID) vs CON | 1 | No concerns | Some concerns | No concerns | Major concerns | Some concerns | No concerns | Low |
| S-EXE (QW) vs CON | 2 | Some concerns | Some concerns | No concerns | Major concerns | No concerns | No concerns | Low |
| S-LIR (QD) vs CON | 3 | No concerns | Some concerns | No concerns | No concerns | Major concerns | No concerns | Moderate |
| S-SEM (QW) vs CON | 3 | No concerns | Some concerns | No concerns | No concerns | No concerns | No concerns | Moderate |
| S-EXE (BID) vs S-EXE (QW) | 0 | No concerns | Some concerns | No concerns | Major concerns | No concerns | Some concerns | Very low |
| S-EXE (BID) vs S-LIR (QD) | 0 | No concerns | Some concerns | No concerns | Major concerns | No concerns | Some concerns | Very low |
| S-EXE (BID) vs S-SEM (QW) | 0 | No concerns | Some concerns | No concerns | Major concerns | No concerns | Some concerns | Very low |
| S-EXE (QW) vs S-LIR (QD) | 0 | No concerns | Some concerns | No concerns | Major concerns | No concerns | Some concerns | Very low |
| S-EXE (QW) vs S-SEM (QW) | 0 | No concerns | Some concerns | No concerns | No concerns | Major concerns | Some concerns | Very low |
| S-LIR (QD) vs S-SEM (QW) | 0 | No concerns | Some concerns | No concerns | Some concerns | Some concerns | Some concerns | Very low |

MID (converted to SMD): 3.0 kg (0.40)

We defined a minimal important difference corresponding to a 3.0 kg reduction in body weight, based on the 5% weight loss recommended by major clinical guidelines (e.g., WHO, ADA, NICE). This threshold reflects an approximate conversion assuming a baseline body weight of 70 kg (i.e., 3.5 kg), and was conservatively rounded to 3.0 kg. This value was then used as the minimal important difference in kilograms for the present analysis.

American Diabetes Association Professional Practice Committee. 8. Obesity and Weight Management for the Prevention and Treatment of Type 2 Diabetes: Standards of Care in Diabetes—2024 (or 2025/2026). Diabetes Care.

National Institute for Health and Care Excellence (NICE). Obesity: identification, assessment and management [CG189]. (Updated 2022/2023).

World Health Organization. (2000). Obesity: preventing and managing the global epidemic: report of a WHO consultation. World Health Organization Technical Report Series, 894.

**Meta-regression analysis**

| Potential moderators | β (SE) |
| --- | --- |
| Proportion of females (k = 9) | 0.03 (0.03) |
| Mean age (k = 8) | 0.03 (0.14) |
| Body weight at baseline (k = 9) | 0.00 (0.05) |
| Baseline glycemic status (studies involving patients without prediabetes or dysglycemia vs. those with these conditions) (k = 9) | -1.05 (0.70) |
| Study duration (k = 9) | -0.06 (0.04) |
| Total participants (k = 9) | 0.00 (0.00) |
| Funding source (industry-sponsored vs. non-industry trials) (k = 9) | 0.22 (0.85) |
| Publication year (k = 9) | -0.14 (0.10) |

**Sensitivity analyses**

(1) **A separate dose- and formulation-specific network meta-analysis**

| S-EXE (10 μg, BID) | -0.548 (-1.975, 0.878) | 0.418 (-1.152, 1.987) | -0.003 (-1.409, 1.403) | 0.706 (-0.669, 2.081) | **3.233 (1.290, 5.177)** | -0.797 (-1.939, 0.344) |
| --- | --- | --- | --- | --- | --- | --- |
|  | S-EXE (2.0 mg, QW) | 0.966 (-0.410, 2.342) | 0.545 (-0.641, 1.732) | **1.254 (0.105, 2.404)** | **3.782 (1.991, 5.573)** | -0.249 (-1.105, 0.608) |
|  |  | S-LIR (1.8 mg, QD) | -0.421 (-1.775, 0.934) | 0.288 (-1.033, 1.610) | **2.816 (0.909, 4.722)** | **-1.215 (-2.292, -0.138)** |
|  |  |  | S-LIR (3.0 mg, QD) | 0.709 (-0.415, 1.833) | **3.236 (1.462, 5.011)** | -0.794 (-1.616, 0.027) |
|  |  |  |  | S-SEM (1.0 mg, QW) | **2.527 (0.777, 4.277)** | **-1.503 (-2.270, -0.737)** |
|  |  |  |  |  | S-SEM (2.0 mg, QW) | **-4.030 (-5.604, -2.457)** |
|  |  |  |  |  |  | CON |

**Global heterogeneity**

Between study variance (𝜏^2^): 0.503 (heterogeneity assessment: high)

(2) **Including only RCTs where participants received clozapine and/or olanzapine**

| S-EXE (BID) | 0.188 (-3.898, 4.274) | 0.418 (-3.616, 4.451) | 2.091 (-1.455, 5.638) | -0.797 (-3.662, 2.067) |
| --- | --- | --- | --- | --- |
|  | S-EXE (QW) | 0.230 (-3.839, 4.298) | 1.903 (-1.683, 5.489) | -0.985 (-3.899, 1.929) |
|  |  | S-LIR (QD) | 1.674 (-1.852, 5.200) | -1.215 (-4.054, 1.625) |
|  |  |  | S-SEM (QW) | **-2.889 (-4.979, -0.798)** |
|  |  |  |  | CON |

**Global heterogeneity**

Between study variance (𝜏^2^): 1.432 (heterogeneity assessment: high)

(3) **Excluding RCTs involving patients with first-episode psychosis**

| S-EXE (BID) | -0.524 (-2.511, 1.462) | 0.172 (-1.780, 2.124) | 1.340 (-0.530, 3.210) | -0.797 (-2.402, 0.807) |
| --- | --- | --- | --- | --- |
|  | S-EXE (QW) | 0.696 (-0.919, 2.311) | **1.864 (0.350, 3.379)** | -0.273 (-1.444, 0.898) |
|  |  | S-LIR (QD) | 1.169 (-0.301, 2.638) | -0.969 (-2.081, 0.143) |
|  |  |  | S-SEM (QW) | **-2.137 (-3.098, -1.176)** |
|  |  |  |  | CON |

**Global heterogeneity**

Between study variance (𝜏^2^): 0.764 (heterogeneity assessment: high)

(4) **Excluding RCTs in which more than 50% of the participants had bipolar disorder**

| S-EXE (QW) | 0.787 (-0.894, 2.468) | **1.874 (0.314, 3.434)** | -0.274 (-1.480, 0.931) |
| --- | --- | --- | --- |
|  | S-LIR (QD) | 1.087 (-0.446, 2.621) | -1.061 (-2.232, 0.110) |
|  |  | S-SEM (QW) | **-2.148 (-3.138, -1.158)** |
|  |  |  | CON |

**Global heterogeneity**

Between study variance (𝜏^2^): 0.792 (heterogeneity assessment: high)

**(5) Excluding the study evaluated as having a high overall risk of bias**

| S-EXE (BID) | -1.181 (-3.159, 0.797) | 0.148 (-1.452, 1.749) | 1.283 (-0.337, 2.902) | -0.797 (-2.185, 0.591) |
| --- | --- | --- | --- | --- |
|  | S-EXE (QW) | 1.329 (-0.290, 2.948) | **2.464 (0.826, 4.102)** | 0.384 (-1.026, 1.793) |
|  |  | S-LIR (QD) | 1.134 (-0.020, 2.289) | **-0.946 (-1.743, -0.148)** |
|  |  |  | S-SEM (QW) | **-2.080 (-2.915, -1.245)** |
|  |  |  |  | CON |

**Global heterogeneity**

Between study variance (𝜏^2^): 0.644 (heterogeneity assessment: high)

**Appendix S2. Body mass index**

9 studies, 567 participants

**League table (SMD with 95% CI)**

| S-EXE (BID) | -0.471 (-2.240, 1.298) | -0.258 (-1.900, 1.385) | 1.260 (-0.401, 2.921) | -0.832 (-2.258, 0.594) |
| --- | --- | --- | --- | --- |
|  | S-EXE (QW) | 0.214 (-1.114, 1.541) | **1.731 (0.381, 3.081)** | -0.361 (-1.408, 0.686) |
|  |  | S-LIR (QD) | **1.518 (0.337, 2.698)** | -0.575 (-1.391, 0.242) |
|  |  |  | S-SEM (QW) | **-2.092 (-2.945, -1.240)** |
|  |  |  |  | CON |

A 1.0 kg/m² change in body mass index corresponds to an SMD of 0.40.

**SUCRA**

| S-EXE (BID) | 0.619 |
| --- | --- |
| S-EXE (QW) | 0.495 |
| S-LIR (QD) | 0.560 |
| S-SEM (QW) | 0.981 |
| CON | 0.021 |

**Global heterogeneity**

Between study variance (𝜏^2^): 0.665 (heterogeneity assessment: high)

**Random-effects design-by-treatment interaction model**

χ^2^  statistic: 30.208 (5 degrees of freedom), P value: 1.000

**Local heterogeneity (I^2^) and incoherence (SIDE test)**

|  | I^2^ (%) |
| --- | --- |
| S-EXE (BID) vs CON | na |
| S-EXE (QW) vs CON | 79.0 |
| S-LIR (QD) vs CON | 9.3 |
| S-SEM (QW) vs CON | 91.4 |

**Contribution matrix**

| random SMD | S-EXE (BID) vs CON | S-EXE (QW) vs CON | S-LIR (QD) vs CON | S-SEM (QW) vs CON |
| --- | --- | --- | --- | --- |
| Mixed estimates |  |  |  |  |
| S-EXE (BID) vs CON | S-EXE (BID) vs CON: 100 | S-EXE (QW) vs CON: 0 | S-LIR (QD) vs CON: 0 | S-SEM (QW) vs CON: 0 |
| S-EXE (QW) vs CON | S-EXE (BID) vs CON: 0 | S-EXE (QW) vs CON: 100 | S-LIR (QD) vs CON: 0 | S-SEM (QW) vs CON: 0 |
| S-LIR (QD) vs CON | S-EXE (BID) vs CON: 0 | S-EXE (QW) vs CON: 0 | S-LIR (QD) vs CON: 100 | S-SEM (QW) vs CON: 0 |
| S-SEM (QW) vs CON | S-EXE (BID) vs CON: 0 | S-EXE (QW) vs CON: 0 | S-LIR (QD) vs CON: 0 | S-SEM (QW) vs CON: 100 |
| Indirect estimates |  |  |  |  |
| S-EXE (BID) vs S-EXE (QW) | S-EXE (BID) vs CON: 50 | S-EXE (QW) vs CON: 50 | S-LIR (QD) vs CON: 0 | S-SEM (QW) vs CON: 0 |
| S-EXE (BID) vs S-LIR (QD) | S-EXE (BID) vs CON: 50 | S-EXE (QW) vs CON: 0 | S-LIR (QD) vs CON: 50 | S-SEM (QW) vs CON: 0 |
| S-EXE (BID) vs S-SEM (QW) | S-EXE (BID) vs CON: 50 | S-EXE (QW) vs CON: 0 | S-LIR (QD) vs CON: 0 | S-SEM (QW) vs CON: 50 |
| S-EXE (QW) vs S-LIR (QD) | S-EXE (BID) vs CON: 0 | S-EXE (QW) vs CON: 50 | S-LIR (QD) vs CON: 50 | S-SEM (QW) vs CON: 0 |
| S-EXE (QW) vs S-SEM (QW) | S-EXE (BID) vs CON: 0 | S-EXE (QW) vs CON: 50 | S-LIR (QD) vs CON: 0 | S-SEM (QW) vs CON: 50 |
| S-LIR (QD) vs S-SEM (QW) | S-EXE (BID) vs CON: 0 | S-EXE (QW) vs CON: 0 | S-LIR (QD) vs CON: 50 | S-SEM (QW) vs CON: 50 |

**Funnel plot (all drugs vs. control)**

As none of the comparisons included 10 or more studies, the assessment of publication bias was not performed.

**CINeMA confidence rating**

| Comparison | Number of studies | Within-study bias | Reporting bias | Indirectness | Imprecision | Heterogeneity | Incoherence | Confidence rating |
| --- | --- | --- | --- | --- | --- | --- | --- | --- |
| S-EXE (BID) vs CON | 1 | No concerns | Some concerns | No concerns | Major concerns | Some concerns | No concerns | Low |
| S-EXE (QW) vs CON | 2 | Some concerns | Some concerns | No concerns | Major concerns | No concerns | No concerns | Low |
| S-LIR (QD) vs CON | 3 | No concerns | Some concerns | No concerns | Major concerns | No concerns | No concerns | Moderate |
| S-SEM (QW) vs CON | 3 | No concerns | Some concerns | No concerns | No concerns | No concerns | No concerns | High |
| S-EXE (BID) vs S-EXE (QW) | 0 | No concerns | Some concerns | No concerns | Major concerns | No concerns | Some concerns | Very low |
| S-EXE (BID) vs S-LIR (QD) | 0 | No concerns | Some concerns | No concerns | Major concerns | No concerns | Some concerns | Very low |
| S-EXE (BID) vs S-SEM (QW) | 0 | No concerns | Some concerns | No concerns | Major concerns | No concerns | Some concerns | Very low |
| S-EXE (QW) vs S-LIR (QD) | 0 | No concerns | Some concerns | No concerns | Major concerns | No concerns | Some concerns | Very low |
| S-EXE (QW) vs S-SEM (QW) | 0 | No concerns | Some concerns | No concerns | No concerns | Major concerns | Some concerns | Very low |
| S-LIR (QD) vs S-SEM (QW) | 0 | No concerns | Some concerns | No concerns | No concerns | Major concerns | Some concerns | Very low |

MID (converted to SMD): 1.0 kg/m² (0.40)

A 3.0 kg weight change corresponds to a BMI fluctuation of approximately 1.0 to 1.2 units for an average-sized adult.

**Appendix S3.** **Waist circumference**

8 studies, 536 participants

**League table (SMD with 95% CI)**

| S-EXE (BID) | -0.456 (-1.700, 0.789) | 0.139 (-1.001, 1.279) | 0.450 (-0.734, 1.634) | -0.625 (-1.616, 0.366) |
| --- | --- | --- | --- | --- |
|  | S-EXE (QW) | 0.595 (-0.347, 1.536) | 0.906 (-0.088, 1.899) | -0.170 (-0.922, 0.584) |
|  |  | S-LIR (QD) | 0.311 (-0.549, 1.171) | **-0.764 (-1.329, -0.199)** |
|  |  |  | S-SEM (QW) | **-1.075 (-1.723, -0.427)** |
|  |  |  |  | CON |

A 5.0 cm change in waist circumference corresponds to an SMD of 0.65.

**SUCRA**

| S-EXE (BID) | 0.598 |
| --- | --- |
| S-EXE (QW) | 0.240 |
| S-LIR (QD) | 0.781 |
| S-SEM (QW) | 0.929 |
| CON | 0.009 |

**Global heterogeneity**

Between study variance (𝜏^2^): 0.414 (heterogeneity assessment: high)

**Random-effects design-by-treatment interaction model**

χ^2^  statistic: 13.498 (4 degrees of freedom), P value: 0.009

**Local heterogeneity (I^2^) and incoherence (SIDE test)**

|  | I^2^ (%) |
| --- | --- |
| S-EXE (BID) vs CON | na |
| S-EXE (QW) vs CON | 69.0 |
| S-LIR (QD) vs CON | 56.5 |
| S-SEM (QW) vs CON | 82.4 |

**Contribution matrix**

| random SMD | S-EXE (BID) vs CON | S-EXE (QW) vs CON | S-LIR (QD) vs CON | S-SEM (QW) vs CON |
| --- | --- | --- | --- | --- |
| Mixed estimates |  |  |  |  |
| S-EXE (BID) vs CON | S-EXE (BID) vs CON: 100 | S-EXE (QW) vs CON: 0 | S-LIR (QD) vs CON: 0 | S-SEM (QW) vs CON: 0 |
| S-EXE (QW) vs CON | S-EXE (BID) vs CON: 0 | S-EXE (QW) vs CON: 100 | S-LIR (QD) vs CON: 0 | S-SEM (QW) vs CON: 0 |
| S-LIR (QD) vs CON | S-EXE (BID) vs CON: 0 | S-EXE (QW) vs CON: 0 | S-LIR (QD) vs CON: 100 | S-SEM (QW) vs CON: 0 |
| S-SEM (QW) vs CON | S-EXE (BID) vs CON: 0 | S-EXE (QW) vs CON: 0 | S-LIR (QD) vs CON: 0 | S-SEM (QW) vs CON: 100 |
| Indirect estimates |  |  |  |  |
| S-EXE (BID) vs S-EXE (QW) | S-EXE (BID) vs CON: 50 | S-EXE (QW) vs CON: 50 | S-LIR (QD) vs CON: 0 | S-SEM (QW) vs CON: 0 |
| S-EXE (BID) vs S-LIR (QD) | S-EXE (BID) vs CON: 50 | S-EXE (QW) vs CON: 0 | S-LIR (QD) vs CON: 50 | S-SEM (QW) vs CON: 0 |
| S-EXE (BID) vs S-SEM (QW) | S-EXE (BID) vs CON: 50 | S-EXE (QW) vs CON: 0 | S-LIR (QD) vs CON: 0 | S-SEM (QW) vs CON: 50 |
| S-EXE (QW) vs S-LIR (QD) | S-EXE (BID) vs CON: 0 | S-EXE (QW) vs CON: 50 | S-LIR (QD) vs CON: 50 | S-SEM (QW) vs CON: 0 |
| S-EXE (QW) vs S-SEM (QW) | S-EXE (BID) vs CON: 0 | S-EXE (QW) vs CON: 50 | S-LIR (QD) vs CON: 0 | S-SEM (QW) vs CON: 50 |
| S-LIR (QD) vs S-SEM (QW) | S-EXE (BID) vs CON: 0 | S-EXE (QW) vs CON: 0 | S-LIR (QD) vs CON: 50 | S-SEM (QW) vs CON: 50 |

**Funnel plot (all drugs vs. control)**

As none of the comparisons included 10 or more studies, the assessment of publication bias was not performed.

**CINeMA confidence rating**

| Comparison | Number of studies | Within-study bias | Reporting bias | Indirectness | Imprecision | Heterogeneity | Incoherence | Confidence rating |
| --- | --- | --- | --- | --- | --- | --- | --- | --- |
| S-EXE (BID) vs CON | 1 | No concerns | Some concerns | No concerns | Some concerns | Some concerns | No concerns | Moderate |
| S-EXE (QW) vs CON | 2 | Some concerns | Some concerns | No concerns | Some concerns | No concerns | No concerns | Moderate |
| S-LIR (QD) vs CON | 3 | No concerns | Some concerns | No concerns | No concerns | Major concerns | No concerns | Moderate |
| S-SEM (QW) vs CON | 2 | No concerns | Some concerns | No concerns | No concerns | Major concerns | No concerns | Moderate |
| S-EXE (BID) vs S-EXE (QW) | 0 | No concerns | Some concerns | No concerns | Major concerns | No concerns | Some concerns | Very low |
| S-EXE (BID) vs S-LIR (QD) | 0 | No concerns | Some concerns | No concerns | Major concerns | No concerns | Some concerns | Very low |
| S-EXE (BID) vs S-SEM (QW) | 0 | No concerns | Some concerns | No concerns | Major concerns | No concerns | Some concerns | Very low |
| S-EXE (QW) vs S-LIR (QD) | 0 | No concerns | Some concerns | No concerns | Major concerns | No concerns | Some concerns | Very low |
| S-EXE (QW) vs S-SEM (QW) | 0 | No concerns | Some concerns | No concerns | Some concerns | Some concerns | Some concerns | Very low |
| S-LIR (QD) vs S-SEM (QW) | 0 | No concerns | Some concerns | No concerns | Major concerns | No concerns | Some concerns | Very low |

MID (converted to SMD): 5.0 cm (0.65)

No established minimal important difference for waist circumference has been defined in major clinical guidelines (e.g., WHO, IDF, NICE). Therefore, we adopted a threshold of 5 cm based on typical changes observed in clinical trials and its consistency with risk category shifts.

World Health Organization. (2011). Waist circumference and waist-hip ratio: report of a WHO expert consultation, Geneva, 8-11 December 2008.

Alberti, K. G., Eckel, R. H., Grundy, S. M., et al. (2009). Harmonizing the metabolic syndrome: a joint interim statement of the International Diabetes Federation Task Force on Epidemiology and Prevention; National Heart, Lung, and Blood Institute; American Heart Association; et al. Circulation, 120(16), 1640–1645.

National Institute for Health and Care Excellence (NICE). (2022). Obesity: identification, assessment and management [CG189]. (Updated versions including [NG246], 2025).

**Appendix S4. Fasting plasma glucose**

7 studies, 419 participants

**League table (SMD with 95% CI)**

| S-EXE (QW) | -0.242 (-1.078, 0.594) | 0.397 (-0.349, 1.143) | -0.505 (-1.122, 0.113) |
| --- | --- | --- | --- |
|  | S-LIR (QD) | 0.639 (-0.063, 1.342) | -0.263 (-0.827, 0.301) |
|  |  | S-SEM (QW) | **-0.902 (-1.321, -0.483)** |
|  |  |  | CON |

A 10 mg/dL change in fasting plasma glucose corresponds to an SMD of 0.72.

**SUCRA**

| S-EXE (QW) | 0.511 |
| --- | --- |
| S-LIR (QD) | 0.335 |
| S-SEM (QW) | 0.930 |
| CON | 0.010 |

**Global heterogeneity**

Between study variance (𝜏^2^): 0.272 (heterogeneity assessment: high)

**Random-effects design-by-treatment interaction model**

χ^2^  statistic: 7.741 (4 degrees of freedom), P value: 0.102

**Local heterogeneity (I^2^) and incoherence (SIDE test)**

|  | I^2^ (%) |
| --- | --- |
| S-EXE (QW) vs CON | 27.2 |
| S-LIR (QD) vs CON | 65.0 |
| S-SEM (QW) vs CON | 43.0 |

**Contribution matrix**

| random SMD | S-EXE (QW) vs CON | S-LIR (QD) vs CON | S-SEM (QW) vs CON |
| --- | --- | --- | --- |
| Mixed estimates |  |  |  |
| S-EXE (QW) vs CON | S-EXE (QW) vs CON: 100 | S-LIR (QD) vs CON: 0 | S-SEM (QW) vs CON: 0 |
| S-LIR (QD) vs CON | S-EXE (QW) vs CON: 0 | S-LIR (QD) vs CON: 100 | S-SEM (QW) vs CON: 0 |
| S-SEM (QW) vs CON | S-EXE (QW) vs CON: 0 | S-LIR (QD) vs CON: 0 | S-SEM (QW) vs CON: 100 |
| Indirect estimates |  |  |  |
| S-EXE (QW) vs S-LIR (QD) | S-EXE (QW) vs CON: 50 | S-LIR (QD) vs CON: 50 | S-SEM (QW) vs CON: 0 |
| S-EXE (QW) vs S-SEM (QW) | S-EXE (QW) vs CON: 50 | S-LIR (QD) vs CON: 0 | S-SEM (QW) vs CON: 50 |
| S-LIR (QD) vs S-SEM (QW) | S-EXE (QW) vs CON: 0 | S-LIR (QD) vs CON: 50 | S-SEM (QW) vs CON: 50 |

**Funnel plot (all drugs vs. control)**

As none of the comparisons included 10 or more studies, the assessment of publication bias was not performed.

**CINeMA confidence rating**

| Comparison | Number of studies | Within-study bias | Reporting bias | Indirectness | Imprecision | Heterogeneity | Incoherence | Confidence rating |
| --- | --- | --- | --- | --- | --- | --- | --- | --- |
| S-EXE (QW) vs CON | 2 | Some concerns | Some concerns | No concerns | Some concerns | Some concerns | No concerns | Low |
| S-LIR (QD) vs CON | 2 | No concerns | Some concerns | No concerns | Some concerns | Some concerns | No concerns | Moderate |
| S-SEM (QW) vs CON | 3 | No concerns | Some concerns | No concerns | No concerns | Some concerns | No concerns | Moderate |
| S-EXE (QW) vs S-LIR (QD) | 0 | No concerns | Some concerns | No concerns | Major concerns | No concerns | Some concerns | Very low |
| S-EXE (QW) vs S-SEM (QW) | 0 | No concerns | Some concerns | No concerns | Some concerns | Some concerns | Some concerns | Very low |
| S-LIR (QD) vs S-SEM (QW) | 0 | No concerns | Some concerns | No concerns | Some concerns | Some concerns | Some concerns | Very low |

MID (converted to SMD): 10 mg/dL (0.72)

American Diabetes Association Professional Practice Committee. Standards of Care in Diabetes—2024. Diabetes Care. 2024;47(Suppl 1):S1-S321.

**Appendix S5. Hemoglobin A1c**

8 studies, 516 participants

**League table (SMD with 95% CI)**

| S-EXE (QW) | 0.222 (-0.756, 1.199) | 0.357 (-0.615, 1.329) | -0.694 (-1.476, 0.088) |
| --- | --- | --- | --- |
|  | S-LIR (QD) | 0.135 (-0.688, 0.958) | **-0.916 (-1.502, -0.329)** |
|  |  | S-SEM (QW) | **-1.051 (-1.628, -0.474)** |
|  |  |  | CON |

A 0.3% change in hemoglobin A1c corresponds to an SMD of 0.99.

**SUCRA**

| S-EXE (QW) | 0.409 |
| --- | --- |
| S-LIR (QD) | 0.891 |
| S-SEM (QW) | 0.987 |
| CON | 0.003 |

**Global heterogeneity**

Between study variance (𝜏^2^): 0.433 (heterogeneity assessment: high)

**Random-effects design-by-treatment interaction model**

χ^2^  statistic: 17.103 (5 degrees of freedom), P value: 0.004

**Local heterogeneity (I^2^) and incoherence (SIDE test)**

|  | I^2^ (%) |
| --- | --- |
| S-EXE (QW) vs CON | 64.9 |
| S-LIR (QD) vs CON | 44.5 |
| S-SEM (QW) vs CON | 81.2 |

**Contribution matrix**

| random SMD | S-EXE (QW) vs CON | S-LIR (QD) vs CON | S-SEM (QW) vs CON |
| --- | --- | --- | --- |
| Mixed estimates |  |  |  |
| S-EXE (QW) vs CON | S-EXE (QW) vs CON: 100 | S-LIR (QD) vs CON: 0 | S-SEM (QW) vs CON: 0 |
| S-LIR (QD) vs CON | S-EXE (QW) vs CON: 0 | S-LIR (QD) vs CON: 100 | S-SEM (QW) vs CON: 0 |
| S-SEM (QW) vs CON | S-EXE (QW) vs CON: 0 | S-LIR (QD) vs CON: 0 | S-SEM (QW) vs CON: 100 |
| Indirect estimates |  |  |  |
| S-EXE (QW) vs S-LIR (QD) | S-EXE (QW) vs CON: 50 | S-LIR (QD) vs CON: 50 | S-SEM (QW) vs CON: 0 |
| S-EXE (QW) vs S-SEM (QW) | S-EXE (QW) vs CON: 50 | S-LIR (QD) vs CON: 0 | S-SEM (QW) vs CON: 50 |
| S-LIR (QD) vs S-SEM (QW) | S-EXE (QW) vs CON: 0 | S-LIR (QD) vs CON: 50 | S-SEM (QW) vs CON: 50 |

**Funnel plot (all drugs vs. control)**

As none of the comparisons included 10 or more studies, the assessment of publication bias was not performed.

**CINeMA confidence rating**

| Comparison | Number of studies | Within-study bias | Reporting bias | Indirectness | Imprecision | Heterogeneity | Incoherence | Confidence rating |
| --- | --- | --- | --- | --- | --- | --- | --- | --- |
| S-EXE (QW) vs CON | 2 | Some concerns | Some concerns | No concerns | Some concerns | Some concerns | No concerns | Low |
| S-LIR (QD) vs CON | 3 | No concerns | Some concerns | No concerns | No concerns | Some concerns | No concerns | Moderate |
| S-SEM (QW) vs CON | 3 | No concerns | Some concerns | No concerns | No concerns | Some concerns | No concerns | Moderate |
| S-EXE (QW) vs S-LIR (QD) | 0 | No concerns | Some concerns | No concerns | Major concerns | No concerns | Some concerns | Very low |
| S-EXE (QW) vs S-SEM (QW) | 0 | No concerns | Some concerns | No concerns | Major concerns | No concerns | Some concerns | Very low |
| S-LIR (QD) vs S-SEM (QW) | 0 | No concerns | Some concerns | No concerns | Major concerns | No concerns | Some concerns | Very low |

MID (converted to SMD): 0.3% (0.99)

American Diabetes Association Professional Practice Committee. Standards of Care in Diabetes—2024. Diabetes Care. 2024;47(Suppl 1):S1-S321.

**Appendix S6. Blood total cholesterol**

7 studies, 485 participants

**League table (SMD with 95% CI)**

| S-EXE (QW) | 0.468 (-0.464, 1.400) | -0.028 (-1.006, 0.950) | 0.014 (-0.732, 0.759) |
| --- | --- | --- | --- |
|  | S-LIR (QD) | -0.496 (-1.341, 0.348) | -0.454 (-1.013, 0.105) |
|  |  | S-SEM (QW) | 0.042 (-0.591, 0.675) |
|  |  |  | CON |

A 40 mg/dL change in blood total cholesterol corresponds to an SMD of 0.75.

**SUCRA**

| S-EXE (QW) | 0.392 |
| --- | --- |
| S-LIR (QD) | 0.558 |
| S-SEM (QW) | 0.319 |
| CON | 0.342 |

**Global heterogeneity**

Between study variance (𝜏^2^): 0.411 (heterogeneity assessment: high)

**Random-effects design-by-treatment interaction model**

χ^2^  statistic: 14.230 (4 degrees of freedom), P value: 0.007

**Local heterogeneity (I^2^) and incoherence (SIDE test)**

|  | I^2^ (%) |
| --- | --- |
| S-EXE (QW) vs CON | 0.0 |
| S-LIR (QD) vs CON | 84.4 |
| S-SEM (QW) vs CON | 0.0 |

**Contribution matrix**

| random SMD | S-EXE (QW) vs CON | S-LIR (QD) vs CON | S-SEM (QW) vs CON |
| --- | --- | --- | --- |
| Mixed estimates |  |  |  |
| S-EXE (QW) vs CON | S-EXE (QW) vs CON: 100 | S-LIR (QD) vs CON: 0 | S-SEM (QW) vs CON: 0 |
| S-LIR (QD) vs CON | S-EXE (QW) vs CON: 0 | S-LIR (QD) vs CON: 100 | S-SEM (QW) vs CON: 0 |
| S-SEM (QW) vs CON | S-EXE (QW) vs CON: 0 | S-LIR (QD) vs CON: 0 | S-SEM (QW) vs CON: 100 |
| Indirect estimates |  |  |  |
| S-EXE (QW) vs S-LIR (QD) | S-EXE (QW) vs CON: 50 | S-LIR (QD) vs CON: 50 | S-SEM (QW) vs CON: 0 |
| S-EXE (QW) vs S-SEM (QW) | S-EXE (QW) vs CON: 50 | S-LIR (QD) vs CON: 0 | S-SEM (QW) vs CON: 50 |
| S-LIR (QD) vs S-SEM (QW) | S-EXE (QW) vs CON: 0 | S-LIR (QD) vs CON: 50 | S-SEM (QW) vs CON: 50 |

**Funnel plot (all drugs vs. control)**

As none of the comparisons included 10 or more studies, the assessment of publication bias was not performed.

**CINeMA confidence rating**

| Comparison | Number of studies | Within-study bias | Reporting bias | Indirectness | Imprecision | Heterogeneity | Incoherence | Confidence rating |
| --- | --- | --- | --- | --- | --- | --- | --- | --- |
| S-EXE (QW) vs CON | 2 | Some concerns | Some concerns | No concerns | Some concerns | No concerns | Some concerns | Low |
| S-LIR (QD) vs CON | 3 | No concerns | Some concerns | No concerns | Some concerns | Some concerns | Some concerns | Low |
| S-SEM (QW) vs CON | 2 | No concerns | Some concerns | No concerns | No concerns | No concerns | Some concerns | Moderate |
| S-EXE (QW) vs S-LIR (QD) | 0 | No concerns | Some concerns | No concerns | Major concerns | No concerns | Some concerns | Very low |
| S-EXE (QW) vs S-SEM (QW) | 0 | No concerns | Some concerns | No concerns | Major concerns | No concerns | Some concerns | Very low |
| S-LIR (QD) vs S-SEM (QW) | 0 | No concerns | Some concerns | No concerns | Some concerns | Some concerns | Some concerns | Very low |

MID (converted to SMD): 40 mg/dL (0.75)

For total cholesterol, we defined the MID as 1.0 mmol/L (38.7 mg/dL). This follows the established clinical threshold for LDL cholesterol, where a reduction of 1.0 mmol/L is consistently associated with a significant decrease in major cardiovascular events according to the 2019 ESC/EAS guideliness.

Mach F, et al. 2019 ESC/EAS Guidelines for the management of dyslipidaemias: lipid modification to reduce cardiovascular risk. Eur Heart J. 2020;41(1):111-188.

**Appendix S7. Blood low density lipoprotein cholesterol**

6 studies, 457 participants

**League table (SMD with 95% CI)**

| S-EXE (QW) | 0.686 (-0.341, 1.713) | 0.376 (-0.677, 1.428) | 0.248 (-0.656, 1.152) |
| --- | --- | --- | --- |
|  | S-LIR (QD) | -0.311 (-1.037, 0.416) | -0.438 (-0.925, 0.049) |
|  |  | S-SEM (QW) | -0.127 (-0.666, 0.412) |
|  |  |  | CON |

A 40 mg/dL change in blood low density lipoprotein cholesterol corresponds to an SMD of 2.2.

**SUCRA**

| S-EXE (QW) | 0.209 |
| --- | --- |
| S-LIR (QD) | 0.618 |
| S-SEM (QW) | 0.552 |
| CON | 0.225 |

**Global heterogeneity**

Between study variance (𝜏^2^): 0.335 (heterogeneity assessment: high)

**Random-effects design-by-treatment interaction model**

χ^2^  statistic: 8.866 (3 degrees of freedom), P value: 0.031

**Local heterogeneity (I^2^) and incoherence (SIDE test)**

|  | I^2^ (%) |
| --- | --- |
| S-EXE (QW) vs CON | na |
| S-LIR (QD) vs CON | 74.7 |
| S-SEM (QW) vs CON | 0.0 |

**Contribution matrix**

| random SMD | S-EXE (QW) vs CON | S-LIR (QD) vs CON | S-SEM (QW) vs CON |
| --- | --- | --- | --- |
| Mixed estimates |  |  |  |
| S-EXE (QW) vs CON | S-EXE (QW) vs CON: 100 | S-LIR (QD) vs CON: 0 | S-SEM (QW) vs CON: 0 |
| S-LIR (QD) vs CON | S-EXE (QW) vs CON: 0 | S-LIR (QD) vs CON: 100 | S-SEM (QW) vs CON: 0 |
| S-SEM (QW) vs CON | S-EXE (QW) vs CON: 0 | S-LIR (QD) vs CON: 0 | S-SEM (QW) vs CON: 100 |
| Indirect estimates |  |  |  |
| S-EXE (QW) vs S-LIR (QD) | S-EXE (QW) vs CON: 50 | S-LIR (QD) vs CON: 50 | S-SEM (QW) vs CON: 0 |
| S-EXE (QW) vs S-SEM (QW) | S-EXE (QW) vs CON: 50 | S-LIR (QD) vs CON: 0 | S-SEM (QW) vs CON: 50 |
| S-LIR (QD) vs S-SEM (QW) | S-EXE (QW) vs CON: 0 | S-LIR (QD) vs CON: 50 | S-SEM (QW) vs CON: 50 |

**Funnel plot (all drugs vs. control)**

As none of the comparisons included 10 or more studies, the assessment of publication bias was not performed.

**CINeMA confidence rating**

| Comparison | Number of studies | Within-study bias | Reporting bias | Indirectness | Imprecision | Heterogeneity | Incoherence | Confidence rating |
| --- | --- | --- | --- | --- | --- | --- | --- | --- |
| S-EXE (QW) vs CON | 1 | No concerns | Some concerns | No concerns | No concerns | Some concerns | No concerns | Moderate |
| S-LIR (QD) vs CON | 3 | No concerns | Some concerns | No concerns | No concerns | Some concerns | No concerns | Moderate |
| S-SEM (QW) vs CON | 2 | No concerns | Some concerns | No concerns | No concerns | No concerns | No concerns | High |
| S-EXE (QW) vs S-LIR (QD) | 0 | No concerns | Some concerns | No concerns | Some concerns | Some concerns | Some concerns | Very low |
| S-EXE (QW) vs S-SEM (QW) | 0 | No concerns | Some concerns | No concerns | Some concerns | Some concerns | Some concerns | Very low |
| S-LIR (QD) vs S-SEM (QW) | 0 | No concerns | Some concerns | No concerns | No concerns | Major concerns | Some concerns | Very low |

MID (converted to SMD): 40 mg/dL ()

For LDL cholesterol, the MID was defined as 38.7 mg/dL (1.0 mmol/L), a threshold established by the CTT meta-analysis and the 2019 ESC/EAS guidelines as being associated with a significant reduction in major cardiovascular events.

Mach F, et al. 2019 ESC/EAS Guidelines for the management of dyslipidaemias: lipid modification to reduce cardiovascular risk. Eur Heart J. 2020;41(1):111-188.

**Appendix S8. Blood high density lipoprotein cholesterol**

7 studies, 419 participants

**League table (SMD with 95% CI)**

| S-EXE (QW) | -0.218 (-1.044, 0.607) | 0.092 (-0.640, 0.824) | 0.171 (-0.436, 0.779) |
| --- | --- | --- | --- |
|  | S-LIR (QD) | 0.310 (-0.382, 1.003) | 0.390 (-0.169, 0.949) |
|  |  | S-SEM (QW) | 0.079 (-0.330, 0.488) |
|  |  |  | CON |

A 5 mg/dL change in blood high density lipoprotein cholesterol corresponds to an SMD of 1.0.

**SUCRA**

| S-EXE (QW) | 0.441 |
| --- | --- |
| S-LIR (QD) | 0.559 |
| S-SEM (QW) | 0.359 |
| CON | 0.175 |

**Global heterogeneity**

Between study variance (𝜏^2^): 0.268 (heterogeneity assessment: high)

**Random-effects design-by-treatment interaction model**

χ^2^  statistic: 7.854 (4 degrees of freedom), P value: 0.097

**Local heterogeneity (I^2^) and incoherence (SIDE test)**

|  | I^2^ (%) |
| --- | --- |
| S-EXE (QW) vs CON | 0.0 |
| S-LIR (QD) vs CON | 0.0 |
| S-SEM (QW) vs CON | 71.6 |

**Contribution matrix**

| random SMD | S-EXE (QW) vs CON | S-LIR (QD) vs CON | S-SEM (QW) vs CON |
| --- | --- | --- | --- |
| Mixed estimates |  |  |  |
| S-EXE (QW) vs CON | S-EXE (QW) vs CON: 100 | S-LIR (QD) vs CON: 0 | S-SEM (QW) vs CON: 0 |
| S-LIR (QD) vs CON | S-EXE (QW) vs CON: 0 | S-LIR (QD) vs CON: 100 | S-SEM (QW) vs CON: 0 |
| S-SEM (QW) vs CON | S-EXE (QW) vs CON: 0 | S-LIR (QD) vs CON: 0 | S-SEM (QW) vs CON: 100 |
| Indirect estimates |  |  |  |
| S-EXE (QW) vs S-LIR (QD) | S-EXE (QW) vs CON: 50 | S-LIR (QD) vs CON: 50 | S-SEM (QW) vs CON: 0 |
| S-EXE (QW) vs S-SEM (QW) | S-EXE (QW) vs CON: 50 | S-LIR (QD) vs CON: 0 | S-SEM (QW) vs CON: 50 |
| S-LIR (QD) vs S-SEM (QW) | S-EXE (QW) vs CON: 0 | S-LIR (QD) vs CON: 50 | S-SEM (QW) vs CON: 50 |

**Funnel plot (all drugs vs. control)**

As none of the comparisons included 10 or more studies, the assessment of publication bias was not performed.

**CINeMA confidence rating**

| Comparison | Number of studies | Within-study bias | Reporting bias | Indirectness | Imprecision | Heterogeneity | Incoherence | Confidence rating |
| --- | --- | --- | --- | --- | --- | --- | --- | --- |
| S-EXE (QW) vs CON | 2 | Some concerns | Some concerns | No concerns | No concerns | Some concerns | No concerns | Moderate |
| S-LIR (QD) vs CON | 2 | No concerns | Some concerns | No concerns | No concerns | Some concerns | No concerns | Moderate |
| S-SEM (QW) vs CON | 3 | No concerns | Some concerns | No concerns | No concerns | Major concerns | No concerns | Moderate |
| S-EXE (QW) vs S-LIR (QD) | 0 | No concerns | Some concerns | No concerns | Major concerns | No concerns | Some concerns | Very low |
| S-EXE (QW) vs S-SEM (QW) | 0 | No concerns | Some concerns | No concerns | Major concerns | No concerns | Some concerns | Very low |
| S-LIR (QD) vs S-SEM (QW) | 0 | No concerns | Some concerns | No concerns | Some concerns | Some concerns | Some concerns | Very low |

MID (converted to SMD): 5 mg/dL (1.0)

HDL cholesterol is considered a marker of cardiovascular risk in major clinical guidelines (e.g., ESC/EAS, ACC/AHA), but is not regarded as a primary treatment target. As no minimal important difference has been defined, thresholds were based on prior clinical literature.

Mach F, et al. 2019 ESC/EAS Guidelines for the management of dyslipidaemias: lipid modification to reduce cardiovascular risk. Eur Heart J. 2020;41(1):111-188.

Catapano AL, et al. 2016 ESC/EAS Guidelines for the Management of Dyslipidaemias. Eur Heart J. 2016;37(39):2999-3058.

Baigent C, et al. Efficacy and safety of more intensive lowering of LDL cholesterol: a meta-analysis of data from 170,000 participants in 26 randomised trials. Lancet. 2010;376(9753):1670-1681.

**Appendix S9. Blood triglycerides**

6 studies, 391 participants

**League table (SMD with 95% CI)**

| S-EXE (QW) | 0.456 (-0.314, 1.226) | 0.326 (-0.371, 1.023) | 0.285 (-0.358, 0.929) |
| --- | --- | --- | --- |
|  | S-LIR (QD) | -0.130 (-0.630, 0.371) | -0.170 (-0.594, 0.253) |
|  |  | S-SEM (QW) | -0.041 (-0.308, 0.227) |
|  |  |  | CON |

A 45 mg/dL change in blood triglycerides corresponds to an SMD of 0.52.

**SUCRA**

| S-EXE (QW) | 0.113 |
| --- | --- |
| S-LIR (QD) | 0.487 |
| S-SEM (QW) | 0.329 |
| CON | 0.198 |

**Global heterogeneity**

Between study variance (𝜏^2^): 0.082 (heterogeneity assessment: low to moderate)

**Random-effects design-by-treatment interaction model**

χ^2^  statistic: 3.309 (3 degrees of freedom), P value: 0.346

**Local heterogeneity (I^2^) and incoherence (SIDE test)**

|  | I^2^ (%) |
| --- | --- |
| S-EXE (QW) vs CON | na |
| S-LIR (QD) vs CON | 0.0 |
| S-SEM (QW) vs CON | 31.4 |

**Contribution matrix**

| random SMD | S-EXE (QW) vs CON | S-LIR (QD) vs CON | S-SEM (QW) vs CON |
| --- | --- | --- | --- |
| Mixed estimates |  |  |  |
| S-EXE (QW) vs CON | S-EXE (QW) vs CON: 100 | S-LIR (QD) vs CON: 0 | S-SEM (QW) vs CON: 0 |
| S-LIR (QD) vs CON | S-EXE (QW) vs CON: 0 | S-LIR (QD) vs CON: 100 | S-SEM (QW) vs CON: 0 |
| S-SEM (QW) vs CON | S-EXE (QW) vs CON: 0 | S-LIR (QD) vs CON: 0 | S-SEM (QW) vs CON: 100 |
| Indirect estimates |  |  |  |
| S-EXE (QW) vs S-LIR (QD) | S-EXE (QW) vs CON: 50 | S-LIR (QD) vs CON: 50 | S-SEM (QW) vs CON: 0 |
| S-EXE (QW) vs S-SEM (QW) | S-EXE (QW) vs CON: 50 | S-LIR (QD) vs CON: 0 | S-SEM (QW) vs CON: 50 |
| S-LIR (QD) vs S-SEM (QW) | S-EXE (QW) vs CON: 0 | S-LIR (QD) vs CON: 50 | S-SEM (QW) vs CON: 50 |

**Funnel plot (all drugs vs. control)**

As none of the comparisons included 10 or more studies, the assessment of publication bias was not performed.

**CINeMA confidence rating**

| Comparison | Number of studies | Within-study bias | Reporting bias | Indirectness | Imprecision | Heterogeneity | Incoherence | Confidence rating |
| --- | --- | --- | --- | --- | --- | --- | --- | --- |
| S-EXE (QW) vs CON | 1 | No concerns | Some concerns | No concerns | Some concerns | No concerns | No concerns | Moderate |
| S-LIR (QD) vs CON | 2 | No concerns | Some concerns | No concerns | Some concerns | Some concerns | No concerns | Moderate |
| S-SEM (QW) vs CON | 3 | No concerns | Some concerns | No concerns | No concerns | Some concerns | No concerns | Moderate |
| S-EXE (QW) vs S-LIR (QD) | 0 | No concerns | Some concerns | No concerns | Major concerns | No concerns | Some concerns | Very low |
| S-EXE (QW) vs S-SEM (QW) | 0 | No concerns | Some concerns | No concerns | Major concerns | No concerns | Some concerns | Very low |
| S-LIR (QD) vs S-SEM (QW) | 0 | No concerns | Some concerns | No concerns | Major concerns | No concerns | Some concerns | Very low |

MID (converted to SMD): 45 mg/dL (0.52)

For triglycerides, we defined the MID as 44.3 mg/dL (0.5 mmol/L). This threshold was selected based on clinical evidence suggesting that a reduction of this magnitude is associated with improved metabolic profiles and potential cardiovascular risk reduction, consistent with established lipid management frameworks.

Mach F, et al. 2019 ESC/EAS Guidelines for the management of dyslipidaemias: lipid modification to reduce cardiovascular risk. Eur Heart J. 2020;41(1):111-188.

Bhatt DL, Steg PG, Miller M, et al. Cardiovascular risk reduction with icosapent ethyl for hypertriglyceridemia. N Engl J Med. 2019;380(1):11-22.

**Appendix S10. Systolic blood pressure**

8 studies, 509 participants

**League table (SMD with 95% CI)**

| S-EXE (QW) | 0.068 (-0.510, 0.647) | 0.279 (-0.281, 0.840) | 0.071 (-0.432, 0.575) |
| --- | --- | --- | --- |
|  | S-LIR (QD) | 0.211 (-0.165, 0.587) | 0.003 (-0.282, 0.288) |
|  |  | S-SEM (QW) | -0.208 (-0.454, 0.037) |
|  |  |  | CON |

A 5 mmHg change in systolic blood pressure corresponds to an SMD of 0.39.

**SUCRA**

| S-EXE (QW) | 0.216 |
| --- | --- |
| S-LIR (QD) | 0.319 |
| S-SEM (QW) | 0.502 |
| CON | 0.330 |

**Global heterogeneity**

Between study variance (𝜏^2^): 0.000 (heterogeneity assessment: low)

**Random-effects design-by-treatment interaction model**

χ^2^  statistic: 3.108 (5 degrees of freedom), P value: 0.683

**Local heterogeneity (I^2^) and incoherence (SIDE test)**

|  | I^2^ (%) |
| --- | --- |
| S-EXE (QW) vs CON | 0.0 |
| S-LIR (QD) vs CON | 0.0 |
| S-SEM (QW) vs CON | 0.0 |

**Contribution matrix**

| random SMD | S-EXE (QW) vs CON | S-LIR (QD) vs CON | S-SEM (QW) vs CON |
| --- | --- | --- | --- |
| Mixed estimates |  |  |  |
| S-EXE (QW) vs CON | S-EXE (QW) vs CON: 100 | S-LIR (QD) vs CON: 0 | S-SEM (QW) vs CON: 0 |
| S-LIR (QD) vs CON | S-EXE (QW) vs CON: 0 | S-LIR (QD) vs CON: 100 | S-SEM (QW) vs CON: 0 |
| S-SEM (QW) vs CON | S-EXE (QW) vs CON: 0 | S-LIR (QD) vs CON: 0 | S-SEM (QW) vs CON: 100 |
| Indirect estimates |  |  |  |
| S-EXE (QW) vs S-LIR (QD) | S-EXE (QW) vs CON: 50 | S-LIR (QD) vs CON: 50 | S-SEM (QW) vs CON: 0 |
| S-EXE (QW) vs S-SEM (QW) | S-EXE (QW) vs CON: 50 | S-LIR (QD) vs CON: 0 | S-SEM (QW) vs CON: 50 |
| S-LIR (QD) vs S-SEM (QW) | S-EXE (QW) vs CON: 0 | S-LIR (QD) vs CON: 50 | S-SEM (QW) vs CON: 50 |

**Funnel plot (all drugs vs. control)**

As none of the comparisons included 10 or more studies, the assessment of publication bias was not performed.

**CINeMA confidence rating**

| Comparison | Number of studies | Within-study bias | Reporting bias | Indirectness | Imprecision | Heterogeneity | Incoherence | Confidence rating |
| --- | --- | --- | --- | --- | --- | --- | --- | --- |
| S-EXE (QW) vs CON | 2 | Some concerns | Some concerns | No concerns | Major concerns | No concerns | No concerns | Low |
| S-LIR (QD) vs CON | 3 | No concerns | Some concerns | No concerns | No concerns | No concerns | No concerns | High |
| S-SEM (QW) vs CON | 3 | No concerns | Some concerns | No concerns | Some concerns | No concerns | No concerns | Moderate |
| S-EXE (QW) vs S-LIR (QD) | 0 | No concerns | Some concerns | No concerns | Major concerns | No concerns | Some concerns | Very low |
| S-EXE (QW) vs S-SEM (QW) | 0 | No concerns | Some concerns | No concerns | Major concerns | No concerns | Some concerns | Very low |
| S-LIR (QD) vs S-SEM (QW) | 0 | No concerns | Some concerns | No concerns | Some concerns | Some concerns | Some concerns | Very low |

MID (converted to SMD): 5 mmHg (0.39)

Mancia G, et al. 2023 ESH Guidelines for the management of arterial hypertension. J Hypertens. 2023;41(12):1874-2071.

Unger T, Borghi C, Charchar F, et al. 2020 International Society of Hypertension Global Hypertension Practice Guidelines. Hypertension. 2020;75(6):1334-1357.

**Appendix S11. Diastolic blood pressure**

8 studies, 509 participants

**League table (SMD with 95% CI)**

| S-EXE (QW) | -0.270 (-0.958, 0.419) | -0.163 (-0.839, 0.513) | -0.134 (-0.713, 0.444) |
| --- | --- | --- | --- |
|  | S-LIR (QD) | 0.106 (-0.405, 0.618) | 0.135 (-0.238, 0.508) |
|  |  | S-SEM (QW) | 0.029 (-0.321, 0.379) |
|  |  |  | CON |

A 2 mmHg change in systolic blood pressure corresponds to an SMD of 0.19.

**SUCRA**

| S-EXE (QW) | 0.499 |
| --- | --- |
| S-LIR (QD) | 0.198 |
| S-SEM (QW) | 0.311 |
| CON | 0.300 |

**Global heterogeneity**

Between study variance (𝜏^2^): 0.201 (heterogeneity assessment: moderate to high)

**Random-effects design-by-treatment interaction model**

χ^2^  statistic: 7.872 (5 degrees of freedom), P value: 0.163

**Local heterogeneity (I^2^) and incoherence (SIDE test)**

|  | I^2^ (%) |
| --- | --- |
| S-EXE (QW) vs CON | 46.8 |
| S-LIR (QD) vs CON | 55.9 |
| S-SEM (QW) vs CON | 0.0 |

**Contribution matrix**

| random SMD | S-EXE (QW) vs CON | S-LIR (QD) vs CON | S-SEM (QW) vs CON |
| --- | --- | --- | --- |
| Mixed estimates |  |  |  |
| S-EXE (QW) vs CON | S-EXE (QW) vs CON: 100 | S-LIR (QD) vs CON: 0 | S-SEM (QW) vs CON: 0 |
| S-LIR (QD) vs CON | S-EXE (QW) vs CON: 0 | S-LIR (QD) vs CON: 100 | S-SEM (QW) vs CON: 0 |
| S-SEM (QW) vs CON | S-EXE (QW) vs CON: 0 | S-LIR (QD) vs CON: 0 | S-SEM (QW) vs CON: 100 |
| Indirect estimates |  |  |  |
| S-EXE (QW) vs S-LIR (QD) | S-EXE (QW) vs CON: 50 | S-LIR (QD) vs CON: 50 | S-SEM (QW) vs CON: 0 |
| S-EXE (QW) vs S-SEM (QW) | S-EXE (QW) vs CON: 50 | S-LIR (QD) vs CON: 0 | S-SEM (QW) vs CON: 50 |
| S-LIR (QD) vs S-SEM (QW) | S-EXE (QW) vs CON: 0 | S-LIR (QD) vs CON: 50 | S-SEM (QW) vs CON: 50 |

**Funnel plot (all drugs vs. control)**

As none of the comparisons included 10 or more studies, the assessment of publication bias was not performed.

**CINeMA confidence rating**

| Comparison | Number of studies | Within-study bias | Reporting bias | Indirectness | Imprecision | Heterogeneity | Incoherence | Confidence rating |
| --- | --- | --- | --- | --- | --- | --- | --- | --- |
| S-EXE (QW) vs CON | 2 | Some concerns | Some concerns | No concerns | Major concerns | No concerns | No concerns | Low |
| S-LIR (QD) vs CON | 3 | No concerns | Some concerns | No concerns | Major concerns | No concerns | No concerns | Moderate |
| S-SEM (QW) vs CON | 3 | No concerns | Some concerns | No concerns | Major concerns | No concerns | No concerns | Moderate |
| S-EXE (QW) vs S-LIR (QD) | 0 | No concerns | Some concerns | No concerns | Major concerns | No concerns | Some concerns | Very low |
| S-EXE (QW) vs S-SEM (QW) | 0 | No concerns | Some concerns | No concerns | Major concerns | No concerns | Some concerns | Very low |
| S-LIR (QD) vs S-SEM (QW) | 0 | No concerns | Some concerns | No concerns | Major concerns | No concerns | Some concerns | Very low |

MID (converted to SMD): 2 mmHg (0.19)

Mancia G, et al. 2023 ESH Guidelines for the management of arterial hypertension. J Hypertens. 2023;41(12):1874-2071.

Unger T, Borghi C, Charchar F, et al. 2020 International Society of Hypertension Global Hypertension Practice Guidelines. Hypertension. 2020;75(6):1334-1357.

**Appendix S12. Death**

5 studies, 418 participants

**League table (OR with 95% CI)**

| S-EXE (QW) | 0.504 (0.005, 55.645) | 0.985 (0.010, 97.329) | 1.000 (0.019, 53.888) |
| --- | --- | --- | --- |
|  | S-LIR (QD) | 1.956 (0.066, 57.587) | 1.986 (0.163, 24.141) |
|  |  | S-SEM (QW) | 1.015 (0.104, 9.925) |
|  |  |  | CON |

**SUCRA**

| S-EXE (QW) | 0.555 |
| --- | --- |
| S-LIR (QD) | 0.385 |
| S-SEM (QW) | 0.526 |
| CON | 0.553 |

**Global heterogeneity**

Between study variance (𝜏^2^): 0.000 (heterogeneity assessment: low)

**Random-effects design-by-treatment interaction model**

χ^2^  statistic: 1.105 (2 degrees of freedom), P value: 0.576

**Local heterogeneity (I^2^) and incoherence (SIDE test)**

|  | I^2^ (%) |
| --- | --- |
| S-EXE (QW) vs CON | na |
| S-LIR (QD) vs CON | 0.0 |
| S-SEM (QW) vs CON | 0.0 |

**Contribution matrix**

| random SMD | S-EXE (QW) vs CON | S-LIR (QD) vs CON | S-SEM (QW) vs CON |
| --- | --- | --- | --- |
| Mixed estimates |  |  |  |
| S-EXE (QW) vs CON | S-EXE (QW) vs CON: 100 | S-LIR (QD) vs CON: 0 | S-SEM (QW) vs CON: 0 |
| S-LIR (QD) vs CON | S-EXE (QW) vs CON: 0 | S-LIR (QD) vs CON: 100 | S-SEM (QW) vs CON: 0 |
| S-SEM (QW) vs CON | S-EXE (QW) vs CON: 0 | S-LIR (QD) vs CON: 0 | S-SEM (QW) vs CON: 100 |
| Indirect estimates |  |  |  |
| S-EXE (QW) vs S-LIR (QD) | S-EXE (QW) vs CON: 50 | S-LIR (QD) vs CON: 50 | S-SEM (QW) vs CON: 0 |
| S-EXE (QW) vs S-SEM (QW) | S-EXE (QW) vs CON: 50 | S-LIR (QD) vs CON: 0 | S-SEM (QW) vs CON: 50 |
| S-LIR (QD) vs S-SEM (QW) | S-EXE (QW) vs CON: 0 | S-LIR (QD) vs CON: 50 | S-SEM (QW) vs CON: 50 |

**Funnel plot (all drugs vs. control)**

As none of the comparisons included 10 or more studies, the assessment of publication bias was not performed.

**CINeMA confidence rating**

| Comparison | Number of studies | Within-study bias | Reporting bias | Indirectness | Imprecision | Heterogeneity | Incoherence | Confidence rating |
| --- | --- | --- | --- | --- | --- | --- | --- | --- |
| S-EXE (QW) vs CON | 1 | Major concerns | Some concerns | No concerns | Major concerns | No concerns | No concerns | Low |
| S-LIR (QD) vs CON | 2 | No concerns | Some concerns | No concerns | Major concerns | No concerns | No concerns | Moderate |
| S-SEM (QW) vs CON | 2 | No concerns | Some concerns | No concerns | Major concerns | No concerns | No concerns | Moderate |
| S-EXE (QW) vs S-LIR (QD) | 0 | Some concerns | Some concerns | No concerns | Major concerns | No concerns | Some concerns | Very low |
| S-EXE (QW) vs S-SEM (QW) | 0 | Some concerns | Some concerns | No concerns | Major concerns | No concerns | Some concerns | Very low |
| S-LIR (QD) vs S-SEM (QW) | 0 | No concerns | Some concerns | No concerns | Major concerns | No concerns | Some concerns | Very low |

**Appendix S13. All cause discontinuation**

9 studies, 595 participants

**League table (OR with 95% CI)**

| S-EXE (BID) | 1.053 (0.060, 18.464) | 1.018 (0.103, 10.091) | 2.697 (0.272, 26.744) | 1.429 (0.210, 9.707) |
| --- | --- | --- | --- | --- |
|  | S-EXE (QW) | 0.967 (0.081, 11.482) | 2.562 (0.216, 30.432) | 1.357 (0.161, 11.408) |
|  |  | S-LIR (QD) | 2.650 (0.445, 15.776) | 1.404 (0.398, 4.955) |
|  |  |  | S-SEM (QW) | 0.530 (0.150, 1.870) |
|  |  |  |  | CON |

**SUCRA**

| S-EXE (BID) | 0.333 |
| --- | --- |
| S-EXE (QW) | 0.386 |
| S-LIR (QD) | 0.349 |
| S-SEM (QW) | 0.728 |
| CON | 0.555 |

**Global heterogeneity**

Between study variance (𝜏^2^): 0.796 (heterogeneity assessment: high)

**Random-effects design-by-treatment interaction model**

χ^2^  statistic: 9.512 (5 degrees of freedom), P value: 0.090

**Local heterogeneity (I^2^) and incoherence (SIDE test)**

|  | I^2^ (%) |
| --- | --- |
| S-EXE (BID) vs CON | na |
| S-EXE (QW) vs CON | 0.0 |
| S-LIR (QD) vs CON | 68.3 |
| S-SEM (QW) vs CON | 36.8 |

**Contribution matrix**

| random SMD | S-EXE (BID) vs CON | S-EXE (QW) vs CON | S-LIR (QD) vs CON | S-SEM (QW) vs CON |
| --- | --- | --- | --- | --- |
| Mixed estimates |  |  |  |  |
| S-EXE (BID) vs CON | S-EXE (BID) vs CON: 100 | S-EXE (QW) vs CON: 0 | S-LIR (QD) vs CON: 0 | S-SEM (QW) vs CON: 0 |
| S-EXE (QW) vs CON | S-EXE (BID) vs CON: 0 | S-EXE (QW) vs CON: 100 | S-LIR (QD) vs CON: 0 | S-SEM (QW) vs CON: 0 |
| S-LIR (QD) vs CON | S-EXE (BID) vs CON: 0 | S-EXE (QW) vs CON: 0 | S-LIR (QD) vs CON: 100 | S-SEM (QW) vs CON: 0 |
| S-SEM (QW) vs CON | S-EXE (BID) vs CON: 0 | S-EXE (QW) vs CON: 0 | S-LIR (QD) vs CON: 0 | S-SEM (QW) vs CON: 100 |
| Indirect estimates |  |  |  |  |
| S-EXE (BID) vs S-EXE (QW) | S-EXE (BID) vs CON: 50 | S-EXE (QW) vs CON: 50 | S-LIR (QD) vs CON: 0 | S-SEM (QW) vs CON: 0 |
| S-EXE (BID) vs S-LIR (QD) | S-EXE (BID) vs CON: 50 | S-EXE (QW) vs CON: 0 | S-LIR (QD) vs CON: 50 | S-SEM (QW) vs CON: 0 |
| S-EXE (BID) vs S-SEM (QW) | S-EXE (BID) vs CON: 50 | S-EXE (QW) vs CON: 0 | S-LIR (QD) vs CON: 0 | S-SEM (QW) vs CON: 50 |
| S-EXE (QW) vs S-LIR (QD) | S-EXE (BID) vs CON: 0 | S-EXE (QW) vs CON: 50 | S-LIR (QD) vs CON: 50 | S-SEM (QW) vs CON: 0 |
| S-EXE (QW) vs S-SEM (QW) | S-EXE (BID) vs CON: 0 | S-EXE (QW) vs CON: 50 | S-LIR (QD) vs CON: 0 | S-SEM (QW) vs CON: 50 |
| S-LIR (QD) vs S-SEM (QW) | S-EXE (BID) vs CON: 0 | S-EXE (QW) vs CON: 0 | S-LIR (QD) vs CON: 50 | S-SEM (QW) vs CON: 50 |

**Funnel plot (all drugs vs. control)**

As none of the comparisons included 10 or more studies, the assessment of publication bias was not performed.

**CINeMA confidence rating**

| Comparison | Number of studies | Within-study bias | Reporting bias | Indirectness | Imprecision | Heterogeneity | Incoherence | Confidence rating |
| --- | --- | --- | --- | --- | --- | --- | --- | --- |
| S-EXE (BID) vs CON | 1 | No concerns | Some concerns | No concerns | Major concerns | No concerns | No concerns | Moderate |
| S-EXE (QW) vs CON | 2 | No concerns | Some concerns | No concerns | Major concerns | No concerns | No concerns | Moderate |
| S-LIR (QD) vs CON | 3 | No concerns | Some concerns | No concerns | Major concerns | No concerns | No concerns | Moderate |
| S-SEM (QW) vs CON | 3 | No concerns | Some concerns | No concerns | Major concerns | No concerns | No concerns | Moderate |
| S-EXE (BID) vs S-EXE (QW) | 0 | No concerns | Some concerns | No concerns | Major concerns | No concerns | Some concerns | Very low |
| S-EXE (BID) vs S-LIR (QD) | 0 | No concerns | Some concerns | No concerns | Major concerns | No concerns | Some concerns | Very low |
| S-EXE (BID) vs S-SEM (QW) | 0 | No concerns | Some concerns | No concerns | Major concerns | No concerns | Some concerns | Very low |
| S-EXE (QW) vs S-LIR (QD) | 0 | No concerns | Some concerns | No concerns | Major concerns | No concerns | Some concerns | Very low |
| S-EXE (QW) vs S-SEM (QW) | 0 | No concerns | Some concerns | No concerns | Major concerns | No concerns | Some concerns | Very low |
| S-LIR (QD) vs S-SEM (QW) | 0 | No concerns | Some concerns | No concerns | Major concerns | No concerns | Some concerns | Very low |

**Meta-regression analysis**

| Potential moderators | β (SE) |
| --- | --- |
| Proportion of females (k = 9) | -0.02 (0.03) |
| Mean age (k = 8) | 0.05 (0.11) |
| Body weight at baseline (k = 9) | -0.04 (0.04) |
| Baseline glycemic status (studies involving patients without prediabetes or dysglycemia vs. those with these conditions) (k = 9) | -0.58 (0.78) |
| Study duration (k = 9) | -0.07 (0.03) |
| Total participants (k = 9) | 0.01 (0.01) |
| Funding source (industry-sponsored vs. non-industry trials) (k = 9) | 0.22 (1.05) |
| Publication year (k = 9) | -0.19 (0.11) |

**Sensitivity analyses**

(5) **A separate dose- and formulation-specific network meta-analysis**

| S-EXE (10 μg, BID) | 1.056 (0.054, 20.463) | 0.269 (0.009, 8.087) | 1.470 (0.121, 17.927) | 2.376 (0.197, 28.586) | 4.615 (0.134, 158.745) | 1.429 (0.192, 10.634) |
| --- | --- | --- | --- | --- | --- | --- |
|  | S-EXE (2.0 mg, QW) | 0.254 (0.008, 8.508) | 1.393 (0.099, 19.556) | 2.250 (0.162, 31.203) | 4.371 (0.115, 166.368) | 1.353 (0.153, 11.981) |
|  |  | S-LIR (1.8 mg, QD) | 5.476 (0.240, 125.048) | 8.846 (0.391, 199.917) | 17.184 (0.313, 944.164) | 5.319 (0.340, 83.221) |
|  |  |  | S-LIR (3.0 mg, QD) | 1.616 (0.199, 13.105) | 3.139 (0.119, 82.806) | 0.972 (0.219, 4.316) |
|  |  |  |  | S-SEM (1.0 mg, QW) | 1.943 (0.074, 50.739) | 0.601 (0.138, 2.612) |
|  |  |  |  |  | S-SEM (2.0 mg, QW) | 0.310 (0.017, 5.700) |
|  |  |  |  |  |  | PLA |

**Global heterogeneity**

Between study variance (𝜏^2^): 0.853 (heterogeneity assessment: high)

(6) **Including only RCTs where participants received clozapine and/or olanzapine**

| S-EXE (BID) | 1.429 (0.022, 92.684) | 0.269 (0.022, 3.293) | 1.531 (0.312, 7.498) | 1.429 (0.442, 4.614) |
| --- | --- | --- | --- | --- |
|  | S-EXE (QW) | 0.188 (0.002, 18.267) | 1.071 (0.017, 67.660) | 1.000 (0.018, 54.839) |
|  |  | S-LIR (QD) | 5.698 (0.486, 66.800) | 5.319 (0.580, 48.750) |
|  |  |  | S-SEM (QW) | 0.933 (0.319, 2.729) |
|  |  |  |  | CON |

**Global heterogeneity**

Between study variance (𝜏^2^): 0.191 (heterogeneity assessment: moderate to high)

(7) **Excluding RCTs involving patients with first-episode psychosis**

| S-EXE (BID) | 1.053 (0.059, 18.739) | 1.431 (0.120, 17.124) | 2.701 (0.268, 27.178) | 1.429 (0.208, 9.835) |
| --- | --- | --- | --- | --- |
|  | S-EXE (QW) | 1.359 (0.096, 19.161) | 2.564 (0.214, 30.763) | 1.356 (0.160, 11.488) |
|  |  | S-LIR (QD) | 1.887 (0.252, 14.103) | 0.998 (0.209, 4.755) |
|  |  |  | S-SEM (QW) | 0.529 (0.149, 1.881) |
|  |  |  |  | CON |

**Global heterogeneity**

Between study variance (𝜏^2^): 0.805 (heterogeneity assessment: high)

(8) **Excluding RCTs in which more than 50% of the participants had bipolar disorder**

| S-EXE (QW) | 0.410 (0.050, 3.385) | 2.338 (0.352, 15.540) | 1.392 (0.252, 7.701) |
| --- | --- | --- | --- |
|  | S-LIR (QD) | **5.700 (1.298, 25.023)** | 3.393 (0.986, 11.677) |
|  |  | S-SEM (QW) | 0.595 (0.264, 1.342) |
|  |  |  | CON |

**Global heterogeneity**

Between study variance (𝜏^2^): 0.109 (heterogeneity assessment: moderate to high)

**(5) Excluding the study evaluated as having a high overall risk of bias**

| S-EXE (BID) | 0.952 (0.034, 26.597) | 0.984 (0.082, 11.886) | 2.735 (0.226, 33.056) | 1.429 (0.177, 11.549) |
| --- | --- | --- | --- | --- |
|  | S-EXE (QW) | 1.034 (0.055, 19.263) | 2.872 (0.154, 53.565) | 1.500 (0.112, 20.033) |
|  |  | S-LIR (QD) | 2.779 (0.408, 18.920) | 1.451 (0.374, 5.629) |
|  |  |  | S-SEM (QW) | 0.522 (0.134, 2.029) |
|  |  |  |  | CON |

**Global heterogeneity**

Between study variance (𝜏^2^): 0.903 (heterogeneity assessment: high)

**Appendix S14. Adverse event–related discontinuation**

9 studies, 595 participants

**League table (OR with 95% CI)**

| S-EXE (BID) | 4.306 (0.223, 83.204) | 8.919 (0.664, 119.893) | 3.140 (0.246, 40.117) | 4.143 (0.402, 42.657) |
| --- | --- | --- | --- | --- |
|  | S-EXE (QW) | 2.072 (0.240, 17.884) | 0.729 (0.090, 5.920) | 0.962 (0.155, 5.971) |
|  |  | S-LIR (QD) | 0.352 (0.076, 1.640) | 0.464 (0.148, 1.462) |
|  |  |  | S-SEM (QW) | 1.319 (0.473, 3.680) |
|  |  |  |  | CON |

**SUCRA**

| S-EXE (BID) | 0.100 |
| --- | --- |
| S-EXE (QW) | 0.527 |
| S-LIR (QD) | 0.680 |
| S-SEM (QW) | 0.371 |
| CON | 0.509 |

**Global heterogeneity**

Between study variance (𝜏^2^): 0.000 (heterogeneity assessment: low)

**Random-effects design-by-treatment interaction model**

χ^2^  statistic: 4.350 (5 degrees of freedom), P value: 0.500

**Local heterogeneity (I^2^) and incoherence (SIDE test)**

|  | I^2^ (%) |
| --- | --- |
| S-EXE (BID) vs CON | na |
| S-EXE (QW) vs CON | 0.0 |
| S-LIR (QD) vs CON | 49.4 |
| S-SEM (QW) vs CON | 0.0 |

**Contribution matrix**

| random SMD | S-EXE (BID) vs CON | S-EXE (QW) vs CON | S-LIR (QD) vs CON | S-SEM (QW) vs CON |
| --- | --- | --- | --- | --- |
| Mixed estimates |  |  |  |  |
| S-EXE (BID) vs CON | S-EXE (BID) vs CON: 100 | S-EXE (QW) vs CON: 0 | S-LIR (QD) vs CON: 0 | S-SEM (QW) vs CON: 0 |
| S-EXE (QW) vs CON | S-EXE (BID) vs CON: 0 | S-EXE (QW) vs CON: 100 | S-LIR (QD) vs CON: 0 | S-SEM (QW) vs CON: 0 |
| S-LIR (QD) vs CON | S-EXE (BID) vs CON: 0 | S-EXE (QW) vs CON: 0 | S-LIR (QD) vs CON: 100 | S-SEM (QW) vs CON: 0 |
| S-SEM (QW) vs CON | S-EXE (BID) vs CON: 0 | S-EXE (QW) vs CON: 0 | S-LIR (QD) vs CON: 0 | S-SEM (QW) vs CON: 100 |
| Indirect estimates |  |  |  |  |
| S-EXE (BID) vs S-EXE (QW) | S-EXE (BID) vs CON: 50 | S-EXE (QW) vs CON: 50 | S-LIR (QD) vs CON: 0 | S-SEM (QW) vs CON: 0 |
| S-EXE (BID) vs S-LIR (QD) | S-EXE (BID) vs CON: 50 | S-EXE (QW) vs CON: 0 | S-LIR (QD) vs CON: 50 | S-SEM (QW) vs CON: 0 |
| S-EXE (BID) vs S-SEM (QW) | S-EXE (BID) vs CON: 50 | S-EXE (QW) vs CON: 0 | S-LIR (QD) vs CON: 0 | S-SEM (QW) vs CON: 50 |
| S-EXE (QW) vs S-LIR (QD) | S-EXE (BID) vs CON: 0 | S-EXE (QW) vs CON: 50 | S-LIR (QD) vs CON: 50 | S-SEM (QW) vs CON: 0 |
| S-EXE (QW) vs S-SEM (QW) | S-EXE (BID) vs CON: 0 | S-EXE (QW) vs CON: 50 | S-LIR (QD) vs CON: 0 | S-SEM (QW) vs CON: 50 |
| S-LIR (QD) vs S-SEM (QW) | S-EXE (BID) vs CON: 0 | S-EXE (QW) vs CON: 0 | S-LIR (QD) vs CON: 50 | S-SEM (QW) vs CON: 50 |

**Funnel plot (all drugs vs. control)**

As none of the comparisons included 10 or more studies, the assessment of publication bias was not performed.

**CINeMA confidence rating**

| Comparison | Number of studies | Within-study bias | Reporting bias | Indirectness | Imprecision | Heterogeneity | Incoherence | Confidence rating |
| --- | --- | --- | --- | --- | --- | --- | --- | --- |
| S-EXE (BID) vs CON | 1 | No concerns | Some concerns | No concerns | Major concerns | No concerns | No concerns | Moderate |
| S-EXE (QW) vs CON | 2 | No concerns | Some concerns | No concerns | Major concerns | No concerns | No concerns | Moderate |
| S-LIR (QD) vs CON | 3 | No concerns | Some concerns | No concerns | Major concerns | No concerns | No concerns | Moderate |
| S-SEM (QW) vs CON | 3 | No concerns | Some concerns | No concerns | Major concerns | No concerns | No concerns | Moderate |
| S-EXE (BID) vs S-EXE (QW) | 0 | No concerns | Some concerns | No concerns | Major concerns | No concerns | Some concerns | Very low |
| S-EXE (BID) vs S-LIR (QD) | 0 | No concerns | Some concerns | No concerns | Major concerns | No concerns | Some concerns | Very low |
| S-EXE (BID) vs S-SEM (QW) | 0 | No concerns | Some concerns | No concerns | Major concerns | No concerns | Some concerns | Very low |
| S-EXE (QW) vs S-LIR (QD) | 0 | No concerns | Some concerns | No concerns | Major concerns | No concerns | Some concerns | Very low |
| S-EXE (QW) vs S-SEM (QW) | 0 | No concerns | Some concerns | No concerns | Major concerns | No concerns | Some concerns | Very low |
| S-LIR (QD) vs S-SEM (QW) | 0 | No concerns | Some concerns | No concerns | Major concerns | No concerns | Some concerns | Very low |

**Appendix S15. Serious adverse events**

7 studies, 521 participants

**League table (OR with 95% CI)**

| S-EXE (BID) | 6.152 (0.289, 130.830) | 7.618 (0.549, 105.626) | 3.789 (0.296, 48.419) | 2.636 (0.224, 30.969) |
| --- | --- | --- | --- | --- |
|  | S-EXE (QW) | 1.238 (0.163, 9.432) | 0.616 (0.090, 4.216) | 0.429 (0.070, 2.620) |
|  |  | S-LIR (QD) | 0.497 (0.161, 1.533) | **0.346 (0.138, 0.868)** |
|  |  |  | S-SEM (QW) | 0.696 (0.363, 1.333) |
|  |  |  |  | CON |

**SUCRA**

| S-EXE (BID) | 0.159 |
| --- | --- |
| S-EXE (QW) | 0.611 |
| S-LIR (QD) | 0.787 |
| S-SEM (QW) | 0.595 |
| CON | 0.390 |

**Global heterogeneity**

Between study variance (𝜏^2^): 0.000 (heterogeneity assessment: low)

**Random-effects design-by-treatment interaction model**

χ^2^  statistic: 0.502 (3 degrees of freedom), P value: 0.918

**Local heterogeneity (I^2^) and incoherence (SIDE test)**

|  | I^2^ (%) |
| --- | --- |
| S-EXE (BID) vs CON | na |
| S-EXE (QW) vs CON | na |
| S-LIR (QD) vs CON | 0.0 |
| S-SEM (QW) vs CON | 0.0 |

**League table (RD with 95% CI)**

| S-EXE (BID) | 0.145 (-0.091, 0.381) | 0.145 (-0.005, 0.296) | 0.113 (-0.050, 0.276) | 0.050 (-0.078, 0.178) |
| --- | --- | --- | --- | --- |
|  | S-EXE (QW) | 0.000 (-0.213, 0.214) | -0.032 (-0.255, 0.190) | -0.095 (-0.293, 0.103) |
|  |  | S-LIR (QD) | -0.033 (-0.161, 0.096) | **-0.095 (-0.175, -0.016)**† |
|  |  |  | S-SEM (QW) | -0.063 (-0.164, 0.038) |
|  |  |  |  | CON |

**NNTH (95% CI)**

†-10.5 (-62.5, -5.7)

A negative NNH indicates that the incidence of the event was lower in the treatment group than in the control group.

**Contribution matrix**

| random SMD | S-EXE (BID) vs CON | S-EXE (QW) vs CON | S-LIR (QD) vs CON | S-SEM (QW) vs CON |
| --- | --- | --- | --- | --- |
| Mixed estimates |  |  |  |  |
| S-EXE (BID) vs CON | S-EXE (BID) vs CON: 100 | S-EXE (QW) vs CON: 0 | S-LIR (QD) vs CON: 0 | S-SEM (QW) vs CON: 0 |
| S-EXE (QW) vs CON | S-EXE (BID) vs CON: 0 | S-EXE (QW) vs CON: 100 | S-LIR (QD) vs CON: 0 | S-SEM (QW) vs CON: 0 |
| S-LIR (QD) vs CON | S-EXE (BID) vs CON: 0 | S-EXE (QW) vs CON: 0 | S-LIR (QD) vs CON: 100 | S-SEM (QW) vs CON: 0 |
| S-SEM (QW) vs CON | S-EXE (BID) vs CON: 0 | S-EXE (QW) vs CON: 0 | S-LIR (QD) vs CON: 0 | S-SEM (QW) vs CON: 100 |
| Indirect estimates |  |  |  |  |
| S-EXE (BID) vs S-EXE (QW) | S-EXE (BID) vs CON: 50 | S-EXE (QW) vs CON: 50 | S-LIR (QD) vs CON: 0 | S-SEM (QW) vs CON: 0 |
| S-EXE (BID) vs S-LIR (QD) | S-EXE (BID) vs CON: 50 | S-EXE (QW) vs CON: 0 | S-LIR (QD) vs CON: 50 | S-SEM (QW) vs CON: 0 |
| S-EXE (BID) vs S-SEM (QW) | S-EXE (BID) vs CON: 50 | S-EXE (QW) vs CON: 0 | S-LIR (QD) vs CON: 0 | S-SEM (QW) vs CON: 50 |
| S-EXE (QW) vs S-LIR (QD) | S-EXE (BID) vs CON: 0 | S-EXE (QW) vs CON: 50 | S-LIR (QD) vs CON: 50 | S-SEM (QW) vs CON: 0 |
| S-EXE (QW) vs S-SEM (QW) | S-EXE (BID) vs CON: 0 | S-EXE (QW) vs CON: 50 | S-LIR (QD) vs CON: 0 | S-SEM (QW) vs CON: 50 |
| S-LIR (QD) vs S-SEM (QW) | S-EXE (BID) vs CON: 0 | S-EXE (QW) vs CON: 0 | S-LIR (QD) vs CON: 50 | S-SEM (QW) vs CON: 50 |

**Funnel plot (all drugs vs. control)**

As none of the comparisons included 10 or more studies, the assessment of publication bias was not performed.

**CINeMA confidence rating**

| Comparison | Number of studies | Within-study bias | Reporting bias | Indirectness | Imprecision | Heterogeneity | Incoherence | Confidence rating |
| --- | --- | --- | --- | --- | --- | --- | --- | --- |
| S-EXE (BID) vs CON | 1 | No concerns | Some concerns | No concerns | Major concerns | No concerns | No concerns | Moderate |
| S-EXE (QW) vs CON | 1 | No concerns | Some concerns | No concerns | Major concerns | No concerns | No concerns | Moderate |
| S-LIR (QD) vs CON | 3 | No concerns | Some concerns | No concerns | No concerns | No concerns | No concerns | Moderate |
| S-SEM (QW) vs CON | 2 | No concerns | Some concerns | No concerns | Major concerns | No concerns | No concerns | Moderate |
| S-EXE (BID) vs S-EXE (QW) | 0 | No concerns | Some concerns | No concerns | Major concerns | No concerns | Some concerns | Very low |
| S-EXE (BID) vs S-LIR (QD) | 0 | No concerns | Some concerns | No concerns | Major concerns | No concerns | Some concerns | Very low |
| S-EXE (BID) vs S-SEM (QW) | 0 | No concerns | Some concerns | No concerns | Major concerns | No concerns | Some concerns | Very low |
| S-EXE (QW) vs S-LIR (QD) | 0 | No concerns | Some concerns | No concerns | Major concerns | No concerns | Some concerns | Very low |
| S-EXE (QW) vs S-SEM (QW) | 0 | No concerns | Some concerns | No concerns | Major concerns | No concerns | Some concerns | Very low |
| S-LIR (QD) vs S-SEM (QW) | 0 | No concerns | Some concerns | No concerns | Major concerns | No concerns | Some concerns | Very low |

**Appendix S16. Injection site-related adverse events**

7 studies, 517 participants

**League table (OR with 95% CI)**

| S-EXE (BID) | 4.216 (0.107, 165.554) | 1.996 (0.249, 15.987) | 1.374 (0.212, 8.914) | 1.286 (0.235, 7.030) |
| --- | --- | --- | --- | --- |
|  | S-EXE (QW) | 0.473 (0.015, 15.188) | 0.326 (0.011, 9.252) | 0.305 (0.012, 7.894) |
|  |  | S-LIR (QD) | 0.688 (0.164, 2.886) | 0.644 (0.194, 2.143) |
|  |  |  | S-SEM (QW) | 0.936 (0.428, 2.045) |
|  |  |  |  | CON |

**SUCRA**

| S-EXE (BID) | 0.322 |
| --- | --- |
| S-EXE (QW) | 0.726 |
| S-LIR (QD) | 0.480 |
| S-SEM (QW) | 0.631 |
| CON | 0.498 |

**Global heterogeneity**

Between study variance (𝜏^2^): 0.000 (heterogeneity assessment: low)

**Random-effects design-by-treatment interaction model**

χ^2^  statistic: 2.402 (3 degrees of freedom), P value: 0.493

**Local heterogeneity (I^2^) and incoherence (SIDE test)**

|  | I^2^ (%) |
| --- | --- |
| S-EXE (BID) vs CON | na |
| S-EXE (QW) vs CON | na |
| S-LIR (QD) vs CON | 18.5 |
| S-SEM (QW) vs CON | 0.0 |

**Contribution matrix**

| random SMD | S-EXE (BID) vs CON | S-EXE (QW) vs CON | S-LIR (QD) vs CON | S-SEM (QW) vs CON |
| --- | --- | --- | --- | --- |
| Mixed estimates |  |  |  |  |
| S-EXE (BID) vs CON | S-EXE (BID) vs CON: 100 | S-EXE (QW) vs CON: 0 | S-LIR (QD) vs CON: 0 | S-SEM (QW) vs CON: 0 |
| S-EXE (QW) vs CON | S-EXE (BID) vs CON: 0 | S-EXE (QW) vs CON: 100 | S-LIR (QD) vs CON: 0 | S-SEM (QW) vs CON: 0 |
| S-LIR (QD) vs CON | S-EXE (BID) vs CON: 0 | S-EXE (QW) vs CON: 0 | S-LIR (QD) vs CON: 100 | S-SEM (QW) vs CON: 0 |
| S-SEM (QW) vs CON | S-EXE (BID) vs CON: 0 | S-EXE (QW) vs CON: 0 | S-LIR (QD) vs CON: 0 | S-SEM (QW) vs CON: 100 |
| Indirect estimates |  |  |  |  |
| S-EXE (BID) vs S-EXE (QW) | S-EXE (BID) vs CON: 50 | S-EXE (QW) vs CON: 50 | S-LIR (QD) vs CON: 0 | S-SEM (QW) vs CON: 0 |
| S-EXE (BID) vs S-LIR (QD) | S-EXE (BID) vs CON: 50 | S-EXE (QW) vs CON: 0 | S-LIR (QD) vs CON: 50 | S-SEM (QW) vs CON: 0 |
| S-EXE (BID) vs S-SEM (QW) | S-EXE (BID) vs CON: 50 | S-EXE (QW) vs CON: 0 | S-LIR (QD) vs CON: 0 | S-SEM (QW) vs CON: 50 |
| S-EXE (QW) vs S-LIR (QD) | S-EXE (BID) vs CON: 0 | S-EXE (QW) vs CON: 50 | S-LIR (QD) vs CON: 50 | S-SEM (QW) vs CON: 0 |
| S-EXE (QW) vs S-SEM (QW) | S-EXE (BID) vs CON: 0 | S-EXE (QW) vs CON: 50 | S-LIR (QD) vs CON: 0 | S-SEM (QW) vs CON: 50 |
| S-LIR (QD) vs S-SEM (QW) | S-EXE (BID) vs CON: 0 | S-EXE (QW) vs CON: 0 | S-LIR (QD) vs CON: 50 | S-SEM (QW) vs CON: 50 |

**Funnel plot (all drugs vs. control)**

As none of the comparisons included 10 or more studies, the assessment of publication bias was not performed.

**CINeMA confidence rating**

| Comparison | Number of studies | Within-study bias | Reporting bias | Indirectness | Imprecision | Heterogeneity | Incoherence | Confidence rating |
| --- | --- | --- | --- | --- | --- | --- | --- | --- |
| S-EXE (BID) vs CON | 1 | No concerns | Some concerns | No concerns | Major concerns | No concerns | No concerns | Moderate |
| S-EXE (QW) vs CON | 1 | No concerns | Some concerns | No concerns | Major concerns | No concerns | No concerns | Moderate |
| S-LIR (QD) vs CON | 2 | No concerns | Some concerns | No concerns | Major concerns | No concerns | No concerns | Moderate |
| S-SEM (QW) vs CON | 3 | No concerns | Some concerns | No concerns | Major concerns | No concerns | No concerns | Moderate |
| S-EXE (BID) vs S-EXE (QW) | 0 | No concerns | Some concerns | No concerns | Major concerns | No concerns | Some concerns | Very low |
| S-EXE (BID) vs S-LIR (QD) | 0 | No concerns | Some concerns | No concerns | Major concerns | No concerns | Some concerns | Very low |
| S-EXE (BID) vs S-SEM (QW) | 0 | No concerns | Some concerns | No concerns | Major concerns | No concerns | Some concerns | Very low |
| S-EXE (QW) vs S-LIR (QD) | 0 | No concerns | Some concerns | No concerns | Major concerns | No concerns | Some concerns | Very low |
| S-EXE (QW) vs S-SEM (QW) | 0 | No concerns | Some concerns | No concerns | Major concerns | No concerns | Some concerns | Very low |
| S-LIR (QD) vs S-SEM (QW) | 0 | No concerns | Some concerns | No concerns | Major concerns | No concerns | Some concerns | Very low |

**Appendix S17. Headache**

5 studies, 318 participants

**League table (OR with 95% CI)**

| S-EXE (BID) | 3.112 (0.570, 16.978) | 3.018 (0.254, 35.809) | 3.000 (0.664, 13.564) |
| --- | --- | --- | --- |
|  | S-LIR (QD) | 0.970 (0.118, 7.985) | 0.964 (0.444, 2.095) |
|  |  | S-SEM (QW) | 0.994 (0.140, 7.058) |
|  |  |  | CON |

**SUCRA**

| S-EXE (BID) | 0.235 |
| --- | --- |
| S-LIR (QD) | 0.341 |
| S-SEM (QW) | 0.530 |
| CON | 0.498 |

**Global heterogeneity**

Between study variance (𝜏^2^): 0.000 (heterogeneity assessment: low)

**Random-effects design-by-treatment interaction model**

χ^2^  statistic: 1.661 (2 degrees of freedom), P value: 0.436

**Local heterogeneity (I^2^) and incoherence (SIDE test)**

|  | I^2^ (%) |
| --- | --- |
| S-EXE (BID) vs CON | na |
| S-LIR (QD) vs CON | 0.0 |
| S-SEM (QW) vs CON | 0.0 |

**Contribution matrix**

| random SMD | S-EXE (BID) vs CON | S-LIR (QD) vs CON | S-SEM (QW) vs CON |
| --- | --- | --- | --- |
| Mixed estimates |  |  |  |
| S-EXE (BID) vs CON | S-EXE (BID) vs CON: 100 | S-LIR (QD) vs CON: 0 | S-SEM (QW) vs CON: 0 |
| S-LIR (QD) vs CON | S-EXE (BID) vs CON: 0 | S-LIR (QD) vs CON: 100 | S-SEM (QW) vs CON: 0 |
| S-SEM (QW) vs CON | S-EXE (BID) vs CON: 0 | S-LIR (QD) vs CON: 0 | S-SEM (QW) vs CON: 100 |
| Indirect estimates |  |  |  |
| S-EXE (BID) vs S-LIR (QD) | S-EXE (BID) vs CON: 50 | S-LIR (QD) vs CON: 50 | S-SEM (QW) vs CON: 0 |
| S-EXE (BID) vs S-SEM (QW) | S-EXE (BID) vs CON: 50 | S-LIR (QD) vs CON: 0 | S-SEM (QW) vs CON: 50 |
| S-LIR (QD) vs S-SEM (QW) | S-EXE (BID) vs CON: 0 | S-LIR (QD) vs CON: 50 | S-SEM (QW) vs CON: 50 |

**Funnel plot (all drugs vs. control)**

As none of the comparisons included 10 or more studies, the assessment of publication bias was not performed.

**CINeMA confidence rating**

| Comparison | Number of studies | Within-study bias | Reporting bias | Indirectness | Imprecision | Heterogeneity | Incoherence | Confidence rating |
| --- | --- | --- | --- | --- | --- | --- | --- | --- |
| S-EXE (BID) vs CON | 1 | No concerns | Some concerns | No concerns | Major concerns | No concerns | No concerns | Moderate |
| S-LIR (QD) vs CON | 2 | No concerns | Some concerns | No concerns | Major concerns | No concerns | No concerns | Moderate |
| S-SEM (QW) vs CON | 2 | No concerns | Some concerns | No concerns | Major concerns | No concerns | No concerns | Moderate |
| S-EXE (BID) vs S-LIR (QD) | 0 | No concerns | Some concerns | No concerns | Major concerns | No concerns | Some concerns | Very low |
| S-EXE (BID) vs S-SEM (QW) | 0 | No concerns | Some concerns | No concerns | Major concerns | No concerns | Some concerns | Very low |
| S-LIR (QD) vs S-SEM (QW) | 0 | No concerns | Some concerns | No concerns | Major concerns | No concerns | Some concerns | Very low |

**Appendix S18. Dizziness**

5 studies, 319 participants

**League table (OR with 95% CI)**

| S-EXE (BID) | 0.580 (0.036, 9.442) | 0.404 (0.028, 5.826) | 0.609 (0.052, 7.146) |
| --- | --- | --- | --- |
|  | S-LIR (QD) | 0.697 (0.132, 3.685) | 1.050 (0.283, 3.895) |
|  |  | S-SEM (QW) | 1.506 (0.539, 4.206) |
|  |  |  | CON |

**SUCRA**

| S-EXE (BID) | 0.695 |
| --- | --- |
| S-LIR (QD) | 0.481 |
| S-SEM (QW) | 0.449 |
| CON | 0.610 |

**Global heterogeneity**

Between study variance (𝜏^2^): 0.000 (heterogeneity assessment: low)

**Random-effects design-by-treatment interaction model**

χ^2^  statistic: 1.133 (2 degrees of freedom), P value: 0.568

**Local heterogeneity (I^2^) and incoherence (SIDE test)**

|  | I^2^ (%) |
| --- | --- |
| S-EXE (BID) vs CON | na |
| S-LIR (QD) vs CON | 0.0 |
| S-SEM (QW) vs CON | 0.0 |

**Contribution matrix**

| random SMD | S-EXE (BID) vs CON | S-LIR (QD) vs CON | S-SEM (QW) vs CON |
| --- | --- | --- | --- |
| Mixed estimates |  |  |  |
| S-EXE (BID) vs CON | S-EXE (BID) vs CON: 100 | S-LIR (QD) vs CON: 0 | S-SEM (QW) vs CON: 0 |
| S-LIR (QD) vs CON | S-EXE (BID) vs CON: 0 | S-LIR (QD) vs CON: 100 | S-SEM (QW) vs CON: 0 |
| S-SEM (QW) vs CON | S-EXE (BID) vs CON: 0 | S-LIR (QD) vs CON: 0 | S-SEM (QW) vs CON: 100 |
| Indirect estimates |  |  |  |
| S-EXE (BID) vs S-LIR (QD) | S-EXE (BID) vs CON: 50 | S-LIR (QD) vs CON: 50 | S-SEM (QW) vs CON: 0 |
| S-EXE (BID) vs S-SEM (QW) | S-EXE (BID) vs CON: 50 | S-LIR (QD) vs CON: 0 | S-SEM (QW) vs CON: 50 |
| S-LIR (QD) vs S-SEM (QW) | S-EXE (BID) vs CON: 0 | S-LIR (QD) vs CON: 50 | S-SEM (QW) vs CON: 50 |

**Funnel plot (all drugs vs. control)**

As none of the comparisons included 10 or more studies, the assessment of publication bias was not performed.

**CINeMA confidence rating**

| Comparison | Number of studies | Within-study bias | Reporting bias | Indirectness | Imprecision | Heterogeneity | Incoherence | Confidence rating |
| --- | --- | --- | --- | --- | --- | --- | --- | --- |
| S-EXE (BID) vs CON | 1 | No concerns | Some concerns | No concerns | Major concerns | No concerns | No concerns | Moderate |
| S-LIR (QD) vs CON | 2 | No concerns | Some concerns | No concerns | Major concerns | No concerns | No concerns | Moderate |
| S-SEM (QW) vs CON | 2 | No concerns | Some concerns | No concerns | Major concerns | No concerns | No concerns | Moderate |
| S-EXE (BID) vs S-LIR (QD) | 0 | No concerns | Some concerns | No concerns | Major concerns | No concerns | Some concerns | Very low |
| S-EXE (BID) vs S-SEM (QW) | 0 | No concerns | Some concerns | No concerns | Major concerns | No concerns | Some concerns | Very low |
| S-LIR (QD) vs S-SEM (QW) | 0 | No concerns | Some concerns | No concerns | Major concerns | No concerns | Some concerns | Very low |

**Appendix S19. Nausea**

7 studies, 496 participants

**League table (OR with 95% CI)**

| S-EXE (QW) | 0.654 (0.143, 2.993) | 1.061 (0.233, 4.824) | 1.969 (0.485, 7.991) |
| --- | --- | --- | --- |
|  | S-LIR (QD) | 1.623 (0.710, 3.711) | **3.012 (1.664, 5.453)** |
|  |  | S-SEM (QW) | **1.856 (1.043, 3.301)** |
|  |  |  | CON |

**SUCRA**

| S-EXE (QW) | 0.334 |
| --- | --- |
| S-LIR (QD) | 0.199 |
| S-SEM (QW) | 0.321 |
| CON | 0.894 |

**Global heterogeneity**

Between study variance (𝜏^2^): 0.000 (heterogeneity assessment: low)

**Random-effects design-by-treatment interaction model**

χ^2^  statistic: 2.575 (4 degrees of freedom), P value: 0.631

**Local heterogeneity (I^2^) and incoherence (SIDE test)**

|  | I^2^ (%) |
| --- | --- |
| S-EXE (QW) vs CON | na |
| S-LIR (QD) vs CON | 0.0 |
| S-SEM (QW) vs CON | 9.1 |

**League table (RD with 95% CI)**

| S-EXE (QW) | -0.141 (-0.422, 0.141) | -0.011 (-0.279, 0.258) | 0.122 (-0.125, 0.370) |
| --- | --- | --- | --- |
|  | S-LIR (QD) | 0.130 (-0.040, 0.300) | **0.263 (0.129, 0.398)**† |
|  |  | S-SEM (QW) | **0.133 (0.029, 0.237)**‡ |
|  |  |  | CON |

**NNTH (95% CI)**

† 3.8 (2.5, 7.8)

‡ 7.5 (4.2, 34.5)

**Contribution matrix**

| random SMD | S-EXE (QW) vs CON | S-LIR (QD) vs CON | S-SEM (QW) vs CON |
| --- | --- | --- | --- |
| Mixed estimates |  |  |  |
| S-EXE (QW) vs CON | S-EXE (QW) vs CON: 100 | S-LIR (QD) vs CON: 0 | S-SEM (QW) vs CON: 0 |
| S-LIR (QD) vs CON | S-EXE (QW) vs CON: 0 | S-LIR (QD) vs CON: 100 | S-SEM (QW) vs CON: 0 |
| S-SEM (QW) vs CON | S-EXE (QW) vs CON: 0 | S-LIR (QD) vs CON: 0 | S-SEM (QW) vs CON: 100 |
| Indirect estimates |  |  |  |
| S-EXE (QW) vs S-LIR (QD) | S-EXE (QW) vs CON: 50 | S-LIR (QD) vs CON: 50 | S-SEM (QW) vs CON: 0 |
| S-EXE (QW) vs S-SEM (QW) | S-EXE (QW) vs CON: 50 | S-LIR (QD) vs CON: 0 | S-SEM (QW) vs CON: 50 |
| S-LIR (QD) vs S-SEM (QW) | S-EXE (QW) vs CON: 0 | S-LIR (QD) vs CON: 50 | S-SEM (QW) vs CON: 50 |

**Funnel plot (all drugs vs. control)**

As none of the comparisons included 10 or more studies, the assessment of publication bias was not performed.

**CINeMA confidence rating**

| Comparison | Number of studies | Within-study bias | Reporting bias | Indirectness | Imprecision | Heterogeneity | Incoherence | Confidence rating |
| --- | --- | --- | --- | --- | --- | --- | --- | --- |
| S-EXE (QW) vs CON | 1 | No concerns | Some concerns | No concerns | Major concerns | No concerns | No concerns | Moderate |
| S-LIR (QD) vs CON | 3 | No concerns | Some concerns | No concerns | No concerns | No concerns | No concerns | Moderate |
| S-SEM (QW) vs CON | 3 | No concerns | Some concerns | No concerns | No concerns | No concerns | No concerns | Moderate |
| S-EXE (QW) vs S-LIR (QD) | 0 | No concerns | Some concerns | No concerns | Major concerns | No concerns | Some concerns | Very low |
| S-EXE (QW) vs S-SEM (QW) | 0 | No concerns | Some concerns | No concerns | Major concerns | No concerns | Some concerns | Very low |
| S-LIR (QD) vs S-SEM (QW) | 0 | No concerns | Some concerns | No concerns | Major concerns | No concerns | Some concerns | Very low |

**Appendix S20. Vomiting**

6 studies, 436 participants

**League table (OR with 95% CI)**

| S-EXE (QW) | 0.420 (0.022, 8.014) | 0.303 (0.016, 5.670) | 0.955 (0.056, 16.265) |
| --- | --- | --- | --- |
|  | S-LIR (QD) | 0.721 (0.240, 2.165) | **2.275 (1.010, 5.127)** |
|  |  | S-SEM (QW) | **3.155 (1.505, 6.615)** |
|  |  |  | CON |

**SUCRA**

| S-EXE (QW) | 0.500 |
| --- | --- |
| S-LIR (QD) | 0.268 |
| S-SEM (QW) | 0.231 |
| CON | 0.874 |

**Global heterogeneity**

Between study variance (𝜏^2^): 0.000 (heterogeneity assessment: low)

**Random-effects design-by-treatment interaction model**

χ^2^  statistic: 1.899 (3 degrees of freedom), P value: 0.594

**Local heterogeneity (I^2^) and incoherence (SIDE test)**

|  | I^2^ (%) |
| --- | --- |
| S-EXE (QW) vs CON | na |
| S-LIR (QD) vs CON | 0.0 |
| S-SEM (QW) vs CON | 0.0 |

**League table (RD with 95% CI)**

| S-EXE (QW) | -0.165 (-0.490, 0.160) | -0.173 (-0.465, 0.118) | -0.002 (-0.245, 0.241) |
| --- | --- | --- | --- |
|  | S-LIR (QD) | -0.008 (-0.278, 0.261) | 0.163 (-0.053, 0.379) |
|  |  | S-SEM (QW) | **0.171 (0.010, 0.332)**† |
|  |  |  | CON |

**NNTH (95% CI)**

† 5.8 (3.0, 100.0)

**Contribution matrix**

| random SMD | S-EXE (QW) vs CON | S-LIR (QD) vs CON | S-SEM (QW) vs CON |
| --- | --- | --- | --- |
| Mixed estimates |  |  |  |
| S-EXE (QW) vs CON | S-EXE (QW) vs CON: 100 | S-LIR (QD) vs CON: 0 | S-SEM (QW) vs CON: 0 |
| S-LIR (QD) vs CON | S-EXE (QW) vs CON: 0 | S-LIR (QD) vs CON: 100 | S-SEM (QW) vs CON: 0 |
| S-SEM (QW) vs CON | S-EXE (QW) vs CON: 0 | S-LIR (QD) vs CON: 0 | S-SEM (QW) vs CON: 100 |
| Indirect estimates |  |  |  |
| S-EXE (QW) vs S-LIR (QD) | S-EXE (QW) vs CON: 50 | S-LIR (QD) vs CON: 50 | S-SEM (QW) vs CON: 0 |
| S-EXE (QW) vs S-SEM (QW) | S-EXE (QW) vs CON: 50 | S-LIR (QD) vs CON: 0 | S-SEM (QW) vs CON: 50 |
| S-LIR (QD) vs S-SEM (QW) | S-EXE (QW) vs CON: 0 | S-LIR (QD) vs CON: 50 | S-SEM (QW) vs CON: 50 |

**Funnel plot (all drugs vs. control)**

As none of the comparisons included 10 or more studies, the assessment of publication bias was not performed.

**CINeMA confidence rating**

| Comparison | Number of studies | Within-study bias | Reporting bias | Indirectness | Imprecision | Heterogeneity | Incoherence | Confidence rating |
| --- | --- | --- | --- | --- | --- | --- | --- | --- |
| S-EXE (QW) vs CON | 1 | No concerns | Some concerns | No concerns | Major concerns | No concerns | No concerns | Moderate |
| S-LIR (QD) vs CON | 2 | No concerns | Some concerns | No concerns | No concerns | Major concerns | No concerns | Moderate |
| S-SEM (QW) vs CON | 3 | No concerns | Some concerns | No concerns | No concerns | Major concerns | No concerns | Moderate |
| S-EXE (QW) vs S-LIR (QD) | 0 | No concerns | Some concerns | No concerns | Major concerns | No concerns | Some concerns | Very low |
| S-EXE (QW) vs S-SEM (QW) | 0 | No concerns | Some concerns | No concerns | Major concerns | No concerns | Some concerns | Very low |
| S-LIR (QD) vs S-SEM (QW) | 0 | No concerns | Some concerns | No concerns | Major concerns | No concerns | Some concerns | Very low |

**Appendix S21. Constipation**

6 studies, 451 participants

**League table (OR with 95% CI)**

| S-LIR (QD) | 0.797 (0.244, 2.598) | **3.136 (1.426, 6.897)** |
| --- | --- | --- |
|  | S-SEM (QW) | **3.937 (1.632, 9.499)** |
|  |  | CON |

**SUCRA**

| S-LIR (QD) | 0.265 |
| --- | --- |
| S-SEM (QW) | 0.121 |
| CON | 0.911 |

**Global heterogeneity**

Between study variance (𝜏^2^): 0.293 (heterogeneity assessment: high)

**Random-effects design-by-treatment interaction model**

χ^2^  statistic: 4.666 (4 degrees of freedom), P value: 0.323

**Local heterogeneity (I^2^) and incoherence (SIDE test)**

|  | I^2^ (%) |
| --- | --- |
| S-LIR (QD) vs CON | 7.9 |
| S-SEM (QW) vs CON | 19.8 |

**League table (RD with 95% CI)**

| S-LIR (QD) | 0.034 (-0.112, 0.182) | **0.217 (0.096, 0.337)**† |
| --- | --- | --- |
|  | S-SEM (QW) | **0.182 (0.098, 0.266)**‡ |
|  |  | CON |

**NNTH (95% CI)**

† 4.6 (3.0, 10.4)

‡ 5.5 (3.8, 10.2)

**Contribution matrix**

| random SMD | S-LIR (QD) vs CON | S-SEM (QW) vs CON |
| --- | --- | --- |
| Mixed estimates |  |  |
| S-LIR (QD) vs CON | S-LIR (QD) vs CON: 100 | S-SEM (QW) vs CON: 0 |
| S-SEM (QW) vs CON | S-LIR (QD) vs CON: 0 | S-SEM (QW) vs CON: 100 |
| Indirect estimates |  |  |
| S-LIR (QD) vs S-SEM (QW) | S-LIR (QD) vs CON: 50 | S-SEM (QW) vs CON: 50 |

**Funnel plot (all drugs vs. control)**

As none of the comparisons included 10 or more studies, the assessment of publication bias was not performed.

**CINeMA confidence rating**

| Comparison | Number of studies | Within-study bias | Reporting bias | Indirectness | Imprecision | Heterogeneity | Incoherence | Confidence rating |
| --- | --- | --- | --- | --- | --- | --- | --- | --- |
| S-LIR (QD) vs CON | 3 | No concerns | Some concerns | No concerns | No concerns | No concerns | No concerns | High |
| S-SEM (QW) vs CON | 3 | No concerns | Some concerns | No concerns | No concerns | No concerns | No concerns | High |
| S-LIR (QD) vs S-SEM (QW) | 0 | No concerns | Some concerns | No concerns | Major concerns | No concerns | Some concerns | Very low |

**Appendix S22. Diarrhea**

7 studies, 496 participants

**League table (OR with 95% CI)**

| S-EXE (QW) | 6.382 (0.275, 147.983) | 8.654 (0.374, 200.497) | 13.378 (0.646, 276.912) |
| --- | --- | --- | --- |
|  | S-LIR (QD) | 1.356 (0.416, 4.422) | 2.096 (0.908, 4.842) |
|  |  | S-SEM (QW) | 1.546 (0.671, 3.560) |
|  |  |  | CON |

**SUCRA**

| S-EXE (QW) | 0.020 |
| --- | --- |
| S-LIR (QD) | 0.227 |
| S-SEM (QW) | 0.310 |
| CON | 0.959 |

**Global heterogeneity**

Between study variance (𝜏^2^): 0.331 (heterogeneity assessment: high)

**Random-effects design-by-treatment interaction model**

χ^2^  statistic: 4.899 (4 degrees of freedom), P value: 0.299

**Local heterogeneity (I^2^) and incoherence (SIDE test)**

|  | I^2^ (%) |
| --- | --- |
| S-EXE (QW) vs CON | na |
| S-LIR (QD) vs CON | 11.9 |
| S-SEM (QW) vs CON | 23.7 |

**Contribution matrix**

| random SMD | S-EXE (QW) vs CON | S-LIR (QD) vs CON | S-SEM (QW) vs CON |
| --- | --- | --- | --- |
| Mixed estimates |  |  |  |
| S-EXE (QW) vs CON | S-EXE (QW) vs CON: 100 | S-LIR (QD) vs CON: 0 | S-SEM (QW) vs CON: 0 |
| S-LIR (QD) vs CON | S-EXE (QW) vs CON: 0 | S-LIR (QD) vs CON: 100 | S-SEM (QW) vs CON: 0 |
| S-SEM (QW) vs CON | S-EXE (QW) vs CON: 0 | S-LIR (QD) vs CON: 0 | S-SEM (QW) vs CON: 100 |
| Indirect estimates |  |  |  |
| S-EXE (QW) vs S-LIR (QD) | S-EXE (QW) vs CON: 50 | S-LIR (QD) vs CON: 50 | S-SEM (QW) vs CON: 0 |
| S-EXE (QW) vs S-SEM (QW) | S-EXE (QW) vs CON: 50 | S-LIR (QD) vs CON: 0 | S-SEM (QW) vs CON: 50 |
| S-LIR (QD) vs S-SEM (QW) | S-EXE (QW) vs CON: 0 | S-LIR (QD) vs CON: 50 | S-SEM (QW) vs CON: 50 |

**Funnel plot (all drugs vs. control)**

As none of the comparisons included 10 or more studies, the assessment of publication bias was not performed.

**CINeMA confidence rating**

| Comparison | Number of studies | Within-study bias | Reporting bias | Indirectness | Imprecision | Heterogeneity | Incoherence | Confidence rating |
| --- | --- | --- | --- | --- | --- | --- | --- | --- |
| S-EXE (QW) vs CON | 1 | No concerns | Some concerns | No concerns | Major concerns | No concerns | No concerns | Moderate |
| S-LIR (QD) vs CON | 3 | No concerns | Some concerns | No concerns | Major concerns | No concerns | No concerns | Moderate |
| S-SEM (QW) vs CON | 3 | No concerns | Some concerns | No concerns | Major concerns | No concerns | No concerns | Moderate |
| S-EXE (QW) vs S-LIR (QD) | 0 | No concerns | Some concerns | No concerns | Major concerns | No concerns | Some concerns | Very low |
| S-EXE (QW) vs S-SEM (QW) | 0 | No concerns | Some concerns | No concerns | Major concerns | No concerns | Some concerns | Very low |
| S-LIR (QD) vs S-SEM (QW) | 0 | No concerns | Some concerns | No concerns | Major concerns | No concerns | Some concerns | Very low |

**Appendix S23.** **Psychiatric hospitalization**

6 studies, 486 participants

**League table (OR with 95% CI)**

| S-EXE (BID) | 0.164 (0.003, 10.284) | 4.140 (0.187, 91.423) | 1.996 (0.109, 36.401) | 1.261 (0.075, 21.266) |
| --- | --- | --- | --- | --- |
|  | S-EXE (QW) | 25.227 (0.953, 667.874) | 12.163 (0.550, 268.943) | 7.683 (0.374, 157.906) |
|  |  | S-LIR (QD) | 0.482 (0.115, 2.013) | 0.305 (0.086, 1.077) |
|  |  |  | S-SEM (QW) | 0.632 (0.324, 1.233) |
|  |  |  |  | CON |

**SUCRA**

| S-EXE (BID) | 0.398 |
| --- | --- |
| S-EXE (QW) | 0.111 |
| S-LIR (QD) | 0.699 |
| S-SEM (QW) | 0.679 |
| CON | 0.512 |

**Global heterogeneity**

Between study variance (𝜏^2^): 0.000 (heterogeneity assessment: low)

**Random-effects design-by-treatment interaction model**

χ^2^  statistic: 0.034 (2 degrees of freedom), P value: 0.983

**Local heterogeneity (I^2^) and incoherence (SIDE test)**

|  | I^2^ (%) |
| --- | --- |
| S-EXE (BID) vs CON | na |
| S-EXE (QW) vs CON | na |
| S-LIR (QD) vs CON | 0.0 |
| S-SEM (QW) vs CON | 0.0 |

**Contribution matrix**

| random SMD | S-EXE (BID) vs CON | S-EXE (QW) vs CON | S-LIR (QD) vs CON | S-SEM (QW) vs CON |
| --- | --- | --- | --- | --- |
| Mixed estimates |  |  |  |  |
| S-EXE (BID) vs CON | S-EXE (BID) vs CON: 100 | S-EXE (QW) vs CON: 0 | S-LIR (QD) vs CON: 0 | S-SEM (QW) vs CON: 0 |
| S-EXE (QW) vs CON | S-EXE (BID) vs CON: 0 | S-EXE (QW) vs CON: 100 | S-LIR (QD) vs CON: 0 | S-SEM (QW) vs CON: 0 |
| S-LIR (QD) vs CON | S-EXE (BID) vs CON: 0 | S-EXE (QW) vs CON: 0 | S-LIR (QD) vs CON: 100 | S-SEM (QW) vs CON: 0 |
| S-SEM (QW) vs CON | S-EXE (BID) vs CON: 0 | S-EXE (QW) vs CON: 0 | S-LIR (QD) vs CON: 0 | S-SEM (QW) vs CON: 100 |
| Indirect estimates |  |  |  |  |
| S-EXE (BID) vs S-EXE (QW) | S-EXE (BID) vs CON: 50 | S-EXE (QW) vs CON: 50 | S-LIR (QD) vs CON: 0 | S-SEM (QW) vs CON: 0 |
| S-EXE (BID) vs S-LIR (QD) | S-EXE (BID) vs CON: 50 | S-EXE (QW) vs CON: 0 | S-LIR (QD) vs CON: 50 | S-SEM (QW) vs CON: 0 |
| S-EXE (BID) vs S-SEM (QW) | S-EXE (BID) vs CON: 50 | S-EXE (QW) vs CON: 0 | S-LIR (QD) vs CON: 0 | S-SEM (QW) vs CON: 50 |
| S-EXE (QW) vs S-LIR (QD) | S-EXE (BID) vs CON: 0 | S-EXE (QW) vs CON: 50 | S-LIR (QD) vs CON: 50 | S-SEM (QW) vs CON: 0 |
| S-EXE (QW) vs S-SEM (QW) | S-EXE (BID) vs CON: 0 | S-EXE (QW) vs CON: 50 | S-LIR (QD) vs CON: 0 | S-SEM (QW) vs CON: 50 |
| S-LIR (QD) vs S-SEM (QW) | S-EXE (BID) vs CON: 0 | S-EXE (QW) vs CON: 0 | S-LIR (QD) vs CON: 50 | S-SEM (QW) vs CON: 50 |

**Funnel plot (all drugs vs. control)**

As none of the comparisons included 10 or more studies, the assessment of publication bias was not performed.

**CINeMA confidence rating**

| Comparison | Number of studies | Within-study bias | Reporting bias | Indirectness | Imprecision | Heterogeneity | Incoherence | Confidence rating |
| --- | --- | --- | --- | --- | --- | --- | --- | --- |
| S-EXE (BID) vs CON | 1 | No concerns | Some concerns | No concerns | Major concerns | No concerns | No concerns | Moderate |
| S-EXE (QW) vs CON | 1 | No concerns | Some concerns | No concerns | Major concerns | No concerns | No concerns | Moderate |
| S-LIR (QD) vs CON | 2 | No concerns | Some concerns | No concerns | Major concerns | No concerns | No concerns | Moderate |
| S-SEM (QW) vs CON | 2 | No concerns | Some concerns | No concerns | Major concerns | No concerns | No concerns | Moderate |
| S-EXE (BID) vs S-EXE (QW) | 0 | No concerns | Some concerns | No concerns | Major concerns | No concerns | Some concerns | Very low |
| S-EXE (BID) vs S-LIR (QD) | 0 | No concerns | Some concerns | No concerns | Major concerns | No concerns | Some concerns | Very low |
| S-EXE (BID) vs S-SEM (QW) | 0 | No concerns | Some concerns | No concerns | Major concerns | No concerns | Some concerns | Very low |
| S-EXE (QW) vs S-LIR (QD) | 0 | No concerns | Some concerns | No concerns | Some concerns | No concerns | Some concerns | Very low |
| S-EXE (QW) vs S-SEM (QW) | 0 | No concerns | Some concerns | No concerns | Major concerns | No concerns | Some concerns | Very low |
| S-LIR (QD) vs S-SEM (QW) | 0 | No concerns | Some concerns | No concerns | Major concerns | No concerns | Some concerns | Very low |

**Appendix S24. Overall symptoms**

6 studies, 359 participants

**League table (SMD with 95% CI)**

| S-EXE (QW) | -0.514 (-1.354, 0.326) | 0.124 (-0.412, 0.659) | 0.093 (-0.383, 0.569) |
| --- | --- | --- | --- |
|  | S-LIR (QD) | 0.638 (-0.096, 1.372) | 0.607 (-0.085, 1.300) |
|  |  | S-SEM (QW) | -0.030 (-0.275, 0.214) |
|  |  |  | CON |

A 15 points change in PANSS score corresponds to an SMD of 1.4.

**SUCRA**

| S-EXE (QW) | 0.428 |
| --- | --- |
| S-LIR (QD) | 0.330 |
| S-SEM (QW) | 0.591 |
| CON | 0.362 |

**Global heterogeneity**

Between study variance (𝜏^2^): 0.000 (heterogeneity assessment: low)

**Random-effects design-by-treatment interaction model**

χ^2^  statistic: 0.520 (3 degrees of freedom), P value: 0.914

**Local heterogeneity (I^2^) and incoherence (SIDE test)**

|  | I^2^ (%) |
| --- | --- |
| S-EXE (QW) vs CON | 0.0 |
| S-LIR (QD) vs CON | na |
| S-SEM (QW) vs CON | 0.0 |

**Contribution matrix**

| random SMD | S-EXE (QW) vs CON | S-LIR (QD) vs CON | S-SEM (QW) vs CON |
| --- | --- | --- | --- |
| Mixed estimates |  |  |  |
| S-EXE (QW) vs CON | S-EXE (QW) vs CON: 100 | S-LIR (QD) vs CON: 0 | S-SEM (QW) vs CON: 0 |
| S-LIR (QD) vs CON | S-EXE (QW) vs CON: 0 | S-LIR (QD) vs CON: 100 | S-SEM (QW) vs CON: 0 |
| S-SEM (QW) vs CON | S-EXE (QW) vs CON: 0 | S-LIR (QD) vs CON: 0 | S-SEM (QW) vs CON: 100 |
| Indirect estimates |  |  |  |
| S-EXE (QW) vs S-LIR (QD) | S-EXE (QW) vs CON: 50 | S-LIR (QD) vs CON: 50 | S-SEM (QW) vs CON: 0 |
| S-EXE (QW) vs S-SEM (QW) | S-EXE (QW) vs CON: 50 | S-LIR (QD) vs CON: 0 | S-SEM (QW) vs CON: 50 |
| S-LIR (QD) vs S-SEM (QW) | S-EXE (QW) vs CON: 0 | S-LIR (QD) vs CON: 50 | S-SEM (QW) vs CON: 50 |

**Funnel plot (all drugs vs. control)**

As none of the comparisons included 10 or more studies, the assessment of publication bias was not performed.

**CINeMA confidence rating**

| Comparison | Number of studies | Within-study bias | Reporting bias | Indirectness | Imprecision | Heterogeneity | Incoherence | Confidence rating |
| --- | --- | --- | --- | --- | --- | --- | --- | --- |
| S-EXE (QW) vs CON | 2 | Some concerns | Some concerns | No concerns | Major concerns | No concerns | No concerns | Low |
| S-LIR (QD) vs CON | 1 | No concerns | Some concerns | No concerns | Major concerns | No concerns | No concerns | Moderate |
| S-SEM (QW) vs CON | 3 | No concerns | Some concerns | No concerns | Major concerns | No concerns | No concerns | Moderate |
| S-EXE (QW) vs S-LIR (QD) | 0 | No concerns | Some concerns | No concerns | Major concerns | Some concerns | Some concerns | Very low |
| S-EXE (QW) vs S-SEM (QW) | 0 | No concerns | Some concerns | No concerns | Major concerns | Major concerns | Some concerns | Very low |
| S-LIR (QD) vs S-SEM (QW) | 0 | No concerns | Some concerns | No concerns | Major concerns | No concerns | Some concerns | Very low |

MID (converted to SMD): PANSS 15 points (1.4)

According to the study by Leucht et al. (2009), a reduction of approximately 15.3 points on the PANSS corresponds to “minimal improvement” on the CGI-I scale.

Leucht S, et al. What does the PANSS mean? Theoretical Medicine and Bioethics. 2009;30(4):307-314.
